# CD4^+^ T cells with latent HIV-1 have reduced proliferative responses to T cell receptor stimulation

**DOI:** 10.1084/jem.20231511

**Published:** 2024-01-25

**Authors:** Joshua T. Kufera, Ciara Armstrong, Fengting Wu, Anushka Singhal, Hao Zhang, Jun Lai, Hannah N. Wilkins, Francesco R. Simonetti, Janet D. Siliciano, Robert F. Siliciano

**Affiliations:** 1 Department of Medicine, Johns Hopkins University School of Medicine, Baltimore, MD, USA; 2 Department of Molecular Microbiology and Immunology, Johns Hopkins Bloomberg School of Public Health, Baltimore, MD, USA; 3 Howard Hughes Medical Institute, Baltimore, MD, USA

## Abstract

The latent reservoir for HIV-1 in resting CD4^+^ T cells persists despite antiretroviral therapy as a barrier to cure. The antigen-driven proliferation of infected cells is a major mechanism of reservoir persistence. However, activation through the T cell antigen receptor (TCR) can induce latent proviruses, leading to viral cytopathic effects and immune clearance. In single-cell studies, we show that, relative to uninfected cells or cells with a defective provirus, CD4^+^ T cells with an intact provirus have a profound proliferative defect in response to TCR stimulation. Virion production was observed in only 16.5% of cultures with an intact provirus, but proliferation was reduced even when no virion production was detected. Proliferation was inversely correlated with in vivo clone size. These results may reflect the effects of previous in vivo proliferation and do not support attempts to reduce the reservoir with antiproliferative agents, which may have greater effects on normal T cell responses.

## Teaser

Proliferation of infected T cells drives HIV-1 persistence. At the single-cell level, Kufera et al. demonstrate that, independent of host immune pressure, infected CD4^+^ T cells carrying an intact HIV-1 provirus have reduced proliferative responses.

## Introduction

The latent reservoir for HIV-1 in resting CD4^+^ T cells represents a major barrier to cure due to its persistence in the setting of antiretroviral therapy (ART), evasion of immune recognition, and capacity for proliferative renewal (Bailey et al., 2006; Bui et al., 2017; Cho et al., 2022; Chun et al., 1995, 1997; Crooks et al., 2015; Finzi et al., 1997, 1999; Ho et al., 1995; Hosmane et al., 2017; Lorenzi et al., 2016; Maldarelli et al., 2014; Siliciano et al., 2003; Simonetti et al., 2016; Tobin et al., 2005; Wagner et al., 2014; Wong et al., 1997). The latent reservoir is extremely stable. Very slow decay has been documented during the first 7 years of ART (half-life = 3.7 years) (Cho et al., 2022; Crooks et al., 2015; Peluso et al., 2020; Siliciano et al., 2003). There may also be qualitative changes, including a selection for proviruses that are not readily induced due to their integration sites (Lian et al., 2023) and a selection for cells that are resistant to cell death pathways (Collora et al., 2022; Cummins et al., 2016; Kuo et al., 2018; Ren et al., 2020).

Recent findings suggest that the reservoir does not continue to decay in people on long-term ART, but rather starts to increase slowly after the first 7 years due to infected cell proliferation (McMyn et al., 2023). The stability of the reservoir makes cure solely from reservoir decay extremely unlikely. Underlying the stability of the latent reservoir are complex dynamics involving the proliferation of infected CD4^+^ T cells, which can be driven by homeostatic cytokine signaling, insertional activation of particular host genes, and, in large part, normal responses to antigen (Chomont et al., 2009; Coffin et al., 2021; Collora et al., 2022; Gantner et al., 2020; Maldarelli et al., 2014; Mendoza et al., 2020; Simonetti et al., 2021; Wagner et al., 2014; Wang et al., 2018). Notably, infected cell clones can reach frequencies that are greater than those of most uninfected cell clones (Collora et al., 2022).

Antigen-stimulated T cells divide rapidly to expand the population of cells with the relevant specificity (De Boer et al., 2003; Zajac et al., 1998). In people with HIV-1 (PWH) on long-term ART, most latently infected CD4^+^ T cells with a replication-competent provirus are members of clonal populations (Bailey et al., 2006; Bui et al., 2017; Cho et al., 2022; Hosmane et al., 2017; Lorenzi et al., 2016; Maldarelli et al., 2014; Simonetti et al., 2016; Tobin et al., 2005; Wagner et al., 2014). These cells have arisen from the proliferation of previously infected cells rather than de novo infection (Bui et al., 2017; Hosmane et al., 2017). Therefore, proliferation is a major mechanism of persistence of the latent reservoir. Antigen-driven proliferation is normally followed by a contraction phase, and large clonal populations of HIV-1–infected CD4^+^ T cells wax and wane in vivo (Wang et al., 2018). For some large clones, the continual activation of a small fraction of the cells gives rise to non-suppressible viremia in the detectable range (Halvas et al., 2020; Simonetti et al., 2016; White et al., 2023). However, little is known about the fate of individual infected T cells within clonal cell populations following stimulation through the T cell receptor (TCR).

The latent reservoir is likely established when activated CD4^+^ T cells become infected before or during the transition back to a resting memory state that is non-permissive for viral gene expression (Chun et al., 1995; Shan et al., 2017). In resting CD4^+^ T cells, active forms of inducible host transcription factors required for HIV-1 transcription, including NFκB, NFAT, and PTEFb, are sequestered in the cytoplasm or in inactive complexes so that HIV-1 transcription is blocked (Böhnlein et al., 1988; Brooks et al., 2003; Duh et al., 1989; Fujinaga et al., 1998; Herrmann and Rice, 1993; Isel and Karn, 1999; Liou et al., 2002; Nabel and Baltimore, 1987; Zhu et al., 1997). Upon stimulation, downstream signaling generates active nuclear forms of these factors, inducing a variety of host gene expression programs to prepare the cell for proliferation and effector function. These same transcription factors can reactivate expression of latent HIV-1 proviruses (Böhnlein et al., 1988; Duh et al., 1989; Liou et al., 2002; Nabel and Baltimore, 1987; Zhu et al., 1997). However, this reactivation is stochastic: in vitro mitogen stimulation robustly activates latently infected CD4^+^ T cells but only induces production of infectious virus from a fraction of the cells (Ho et al., 2013; Hosmane et al., 2017; Kwon et al., 2020; Simonetti et al., 2020). Additional rounds of in vitro stimulation can induce additional proviruses, but most remain latent (Ho et al., 2013; Hosmane et al., 2017; Kwon et al., 2020). The factors that determine whether a provirus will be induced following T cell activation remain poorly understood. Even among cells that are part of a clone, induction may differ.

There is also uncertainty about the fate of infected cells following activation through the TCR. Although the same stimuli can induce both viral gene expression and cell proliferation, the relationship between these cell fates is unclear. It remains unknown whether, following induction, an infected cell expressing HIV-1 proteins can survive and proceed to divide. Induction of a latent HIV-1 provirus and subsequent transcription and expression of HIV-1 proteins can enable immune clearance or lead to cell death from viral cytopathic effects. In vivo, most productively infected cells have a very short half-life (∼1 day) (Ho et al., 1995; Wei et al., 1995). Some viral proteins including Env and Tat can trigger apoptosis, while Vpr can cause cell cycle arrest (Chen et al., 2002; He et al., 1995; Laurent-Crawford et al., 1993; Zhang and Bieniasz, 2020). Conversely, cells harboring a provirus that is not induced following T cell activation can evade immune recognition and viral cytopathic effects. This may explain why cells with defective proviruses show no in vivo decay (Bruner et al., 2019; Cho et al., 2022; Gandhi et al., 2021; Peluso et al., 2020).

The integration site of an HIV-1 provirus can affect both host and viral gene transcription, each of which can influence cell fate. Proviral integration into a small subset of cancer- or proliferation-associated genes can affect expression of the host gene in a way that confers a growth or survival advantage for the infected cell (Coffin et al., 2021; Cohn et al., 2015; Ikeda et al., 2007; Maldarelli et al., 2014; Wagner et al., 2014). In addition, the initiation of HIV-1 transcription may be hindered for proviruses present in heterochromatic regions, gene deserts, or satellite DNA, resulting in a survival advantage (Einkauf et al., 2022; Jordan et al., 2003).

The goal of this study was to determine the effects of HIV-1 infection, virion production, and HIV-1 integration sites on CD4^+^ T cell proliferation following cellular activation. Among HIV-1 proviruses, <10% are intact, replication-competent proviruses that have the potential to produce infectious HIV-1 virions (Bruner et al., 2016; Cohn et al., 2015; Hiener et al., 2017; Ho et al., 2013; Imamichi et al., 2016; Li et al., 2015). The rest contain deletions or APOBEC3G-mediated hypermutation that render the virus incapable of replication. We designed and optimized a microculture assay to directly compare the proliferation of uninfected primary CD4^+^ T cells with the proliferation of primary CD4^+^ T cells carrying a defective or genetically intact HIV-1 provirus. In addition to quantifying clonal expansion starting from single HIV-1–infected cells, we measured the number of virions released in each culture well. To better understand the proliferative potential of infected cells in vivo, we identified infected cell clones and compared their in vitro proliferation from a single cell with their frequency in vivo. The single-cell studies described here provide a better understanding of the unique mechanisms that influence the proliferative behavior of HIV-1–infected cells, inform the potential risks and benefits of anti-proliferative strategies, and reveal reservoir vulnerabilities that may be more readily targetable.

## Results

### Single-cell analysis of infected cell responses to TCR stimulation

**Figure 1.**
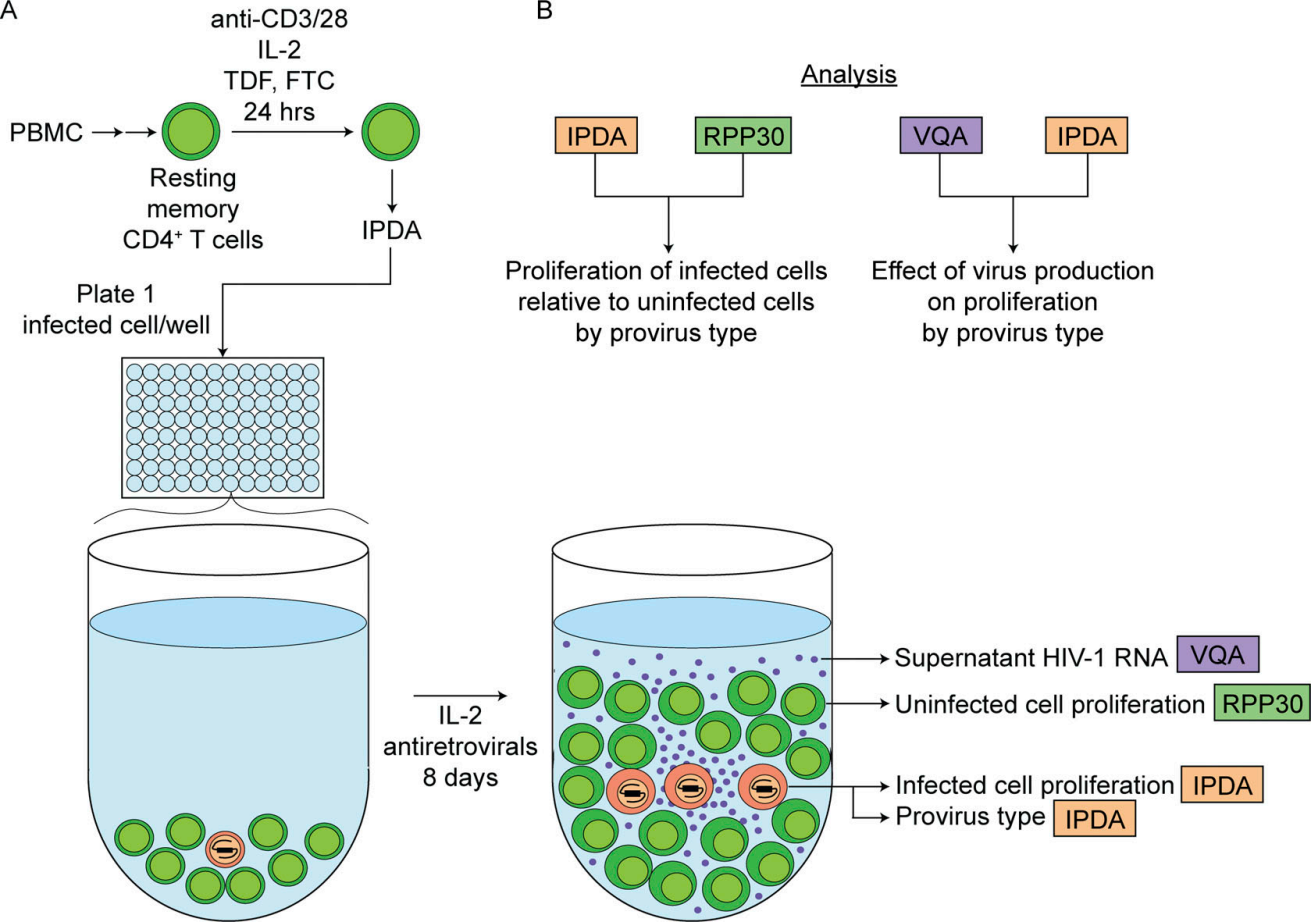
**Single-cell analysis of infected cell responses to TCR stimulation. (A)** Assay method. Resting memory CD4^+^ T cells were stimulated for 24 h with bead-bound anti-CD3 and anti-CD28. IPDA analysis was then used to determine the frequency of infected cells (orange) for plating at limiting dilution. After 7–8 days, proliferation of infected cells and proviral intactness were determined by IPDA analysis of each well. Total cell proliferation in the same wells was determined by ddPCR for the host gene RPP30, and virion production was measured by VQA analysis of culture supernatants. **(B)** Method of analysis. IPDA analysis of each well provides the number of infected cells generated from a single infected cell plated at the beginning of the culture. RPP30 analysis of each well provides a measure of the total number of cells generated by the T cell proliferation that occurs in each well. The vast majority of these are uninfected cells (green), and thus the comparison of IPDA and RPP30 results provides an indication of the proliferative potential of infected cells relative to uninfected CD4^+^ T cells. Because the IPDA also provides an indication of proviral intactness, this comparison can be done for both cells with intact and defective proviruses. Similarly, measurement of supernatant virions in each well allows assessment of the effects of virus production on infected cell proliferation for cells with intact and defective proviruses. See Materials and methods for details and text for references.

To analyze the responses of individual HIV-1–infected CD4^+^ T cells to TCR stimulation, we purified resting memory CD4^+^ T cells from the peripheral blood of study participants on ART and observed the fate of individual infected cells following cellular activation through the TCR (Fig. 1). Resting memory CD4^+^ T cells were examined because the frequency of cells with an intact HIV-1 provirus is approximately three times higher in this subset than in the resting naïve subset (Kwon et al., 2020). In previous studies, we have examined proliferation of infected cells after multiple rounds of in vitro stimulation (Hosmane et al., 2017; Kwon et al., 2020; Bruner et al., 2019). In those cultures, analysis of proliferation is complicated by cell death occurring during long-term culture. Therefore, in the present study, we analyzed proliferation 7–8 days after a single in vitro simulation under optimized conditions.

Isolating individual latently infected CD4^+^ T cells is challenging, as they do not express viral proteins, nor is there any known marker that differentiates them from uninfected resting CD4^+^ T cells (Bertagnolli et al., 2018; Hermankova et al., 2003; Richman et al., 2009). We reasoned that accurate measurement of the frequency of infected cells would allow plating of approximately one infected cell per culture well along with many autologous uninfected cells, thereby enabling a direct comparison between the behavior of the infected cell and uninfected cells within the same culture well. To distribute cells into microtiter plates at appropriate dilutions, we analyzed infected cell frequencies using the intact proviral DNA assay (IPDA), a droplet digital PCR (ddPCR) assay that directly counts infected cells and also provides information on the presence of common fatal proviral defects (Bruner et al., 2019). We used this assay to measure the number of progeny cells generated because it can detect individual proviruses with high sensitivity and has the throughput necessary for the analysis of thousands of microtiter wells. It also provides for discrimination between proviruses that have the most common types of fatal defects, namely large internal deletions and extensive APOBEC3-mediated hypermutation, and proviruses that lack these defects. The latter are termed intact, but importantly with the understanding that a fraction of these proviruses contain defects that could affect viral fitness. This fraction was initially estimated at 30% (Bruner et al., 2019).

Several studies have demonstrated that antigen is an important driver of infected cell proliferation in vivo (Collora et al., 2022; Gantner et al., 2020; Mendoza et al., 2020; Simonetti et al., 2021). Therefore, resting memory CD4^+^ T cells were activated through the TCR using anti-CD3/CD28 microbeads. IL-2 was included in culture medium so that the T cells had the three signals required for T cell activation (Bachmann and Oxenius, 2007; Germain, 1994; Mueller et al., 1989; Steinman, 1991). Following T cell stimulation for 24 h and prior to infected cell proliferation, aliquots of activated CD4^+^ T cells were plated in 96-well plates at or below one infected cell per well based on IPDA analysis of infected cell frequency. Cells were then cultured for 6–7 days in the presence of the antiretroviral drugs emtricitabine (FTC) and tenofovir disoproxil fumarate (TDF) to prevent infection of new cells. The number of progeny cells generated from a single infected cell over the course of the culture was then determined by digital counting using the IPDA. The proliferation of uninfected cells in the same well was quantitated using a ddPCR assay for the host gene RPP30 as described in Materials and methods (Bruner et al., 2019). In addition to measuring infected and uninfected cell proliferation, we determined the number of virions produced in each well using the Viral Quantification Assay (VQA), a previously described quantitative PCR (qPCR) assay specific for the 3′ end of all HIV-1 RNAs (Bullen et al., 2014; Shan et al., 2013). For a representative subset of samples, we analyzed the integration sites and/or near full-length (nFL) sequences of the HIV-1 proviruses to further understand the relationship between proliferation and virus production.

### Analysis of proliferation of infected and uninfected CD4^+^ T cells from PWH

**Figure 2.**
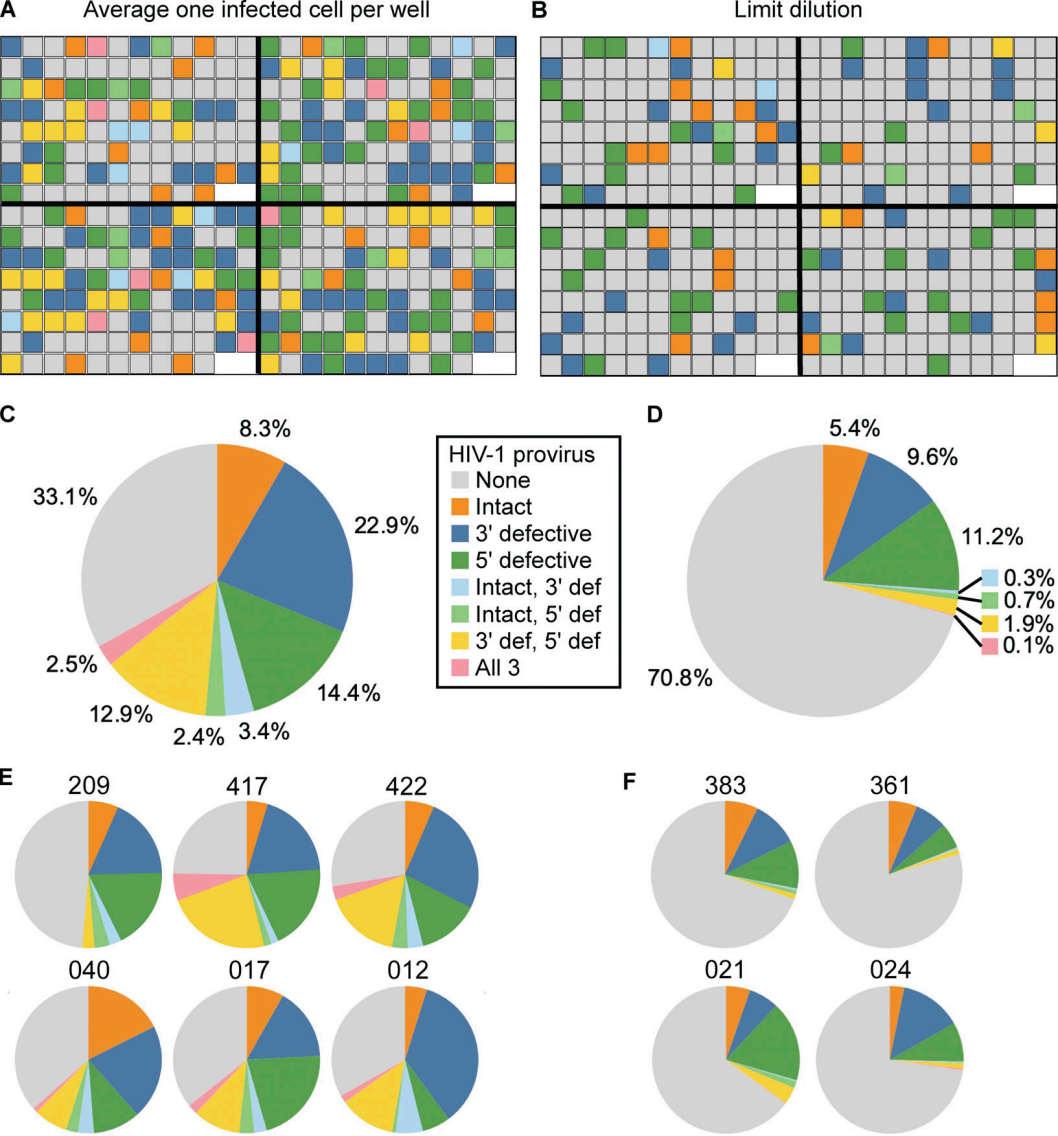
**IPDA analysis of infected cell proliferation. (A)** Map of four representative assay plates from two study participants (left, donor 422; right, donor 017) in which CD4^+^ T cells were distributed into 96-well plates such that each well received on average one infected cell. Colors indicate types of proviruses detected in each well by IPDA analysis, as shown in the key. **(B)** Map of four representative plates from two study participants (left, donor 383; right, donor 021) in which infected cells were further diluted to 0.35 infected cells/well. Colors indicate types of proviruses detected in each well by IPDA analysis. **(C)** Pie chart showing the fraction of microculture wells with each provirus type as determined by IPDA for six participants analyzed with plating scheme 1 (*n* = 1,893 replicate wells). **(D)** Pie chart showing the fraction of microculture wells with each provirus type as determined by IPDA for four participants analyzed with plating scheme 2 (*n* = 2,590 replicate wells). **(E)** Pie charts for individual study participants for plating scheme 1. Numbers indicate participant IDs. **(F)** Pie charts for individual study participants for plating scheme 2.

Using an optimized experimental protocol (see Materials and methods), we analyzed the proliferative responses of infected and uninfected memory CD4^+^ T cells from 10 PWH who were on suppressive ART regimens for a minimum of 10 years (Fig. 2). Characteristics of the study participants are given in Table S1. Resting memory CD4^+^ T cells were purified from peripheral blood (see Materials and methods). After a 24-h cell stimulation with bead-bound antibodies to CD3 and CD28 and initial IPDA analysis, memory CD4^+^ T cells were distributed into 96-well culture plates using two different plating schemes. Scheme 1 was designed to maximize the number of infected cells that could be analyzed. Each well received on average one infected cell. Scheme 2 was designed to provide a high probability that wells initially contained only a single infected cell. In this scheme, infected cells were plated at limit dilution (on average 0.35 infected cells per well). Initial IPDA results and plating statistics for each donor are summarized in Table S2. As discussed below, both plating schemes gave similar results with respect to infected cell proliferation. Wells seeded with more than one infected cell, as evidenced by IPDA analysis of provirus type, were excluded from subsequent analyses where appropriate.

After culturing for a total of 8 days (except if cell density reached 1 million cells/ml on day 7; see Materials and methods), HIV-1 proviruses in each well were digitally counted using the IPDA, while total cells were digitally counted using ddPCR analysis of a diploid host gene, RPP30 (Bruner et al., 2019). In each well, we characterized proviruses as intact, 3′ defective, or 5′ defective using the IPDA (see Materials and methods). The fraction of wells positive for proviruses was very close to values expected based on the initial plating density (Fig. 2, C–F; and Table S3) and was lower for plating scheme 2, as expected. Moreover, the number of wells with more than one type of provirus was much smaller for plating scheme 2 (Fig. 2, D and F). Using Poisson statistics, we confirmed that the goal of isolating single infected cells was achieved in roughly the expected number of culture wells (Fig. 2 and Table S3). This result indicated that the initial IPDA analysis of infected cell frequency was accurate and that the plating method gave the expected number of input infected cells. As discussed in the Materials and methods, we estimate that on average four to five rounds of division are required for detection in the post-culture IPDA based on losses during DNA extraction and the fraction of extracted DNA analyzed by IPDA. A good agreement between the observed and expected number of wells with detectable proviruses (Table S3) indicates that most or all of the infected cells plated went through at least five divisions following TCR stimulation. However, as discussed below, infected cells did not proliferate to the same degree as uninfected CD4^+^ T cells in the same culture wells.

### CD4^+^ T cells with an intact HIV-1 provirus displayed a reduced proliferative response to TCR stimulation in vitro

**Figure 3.**
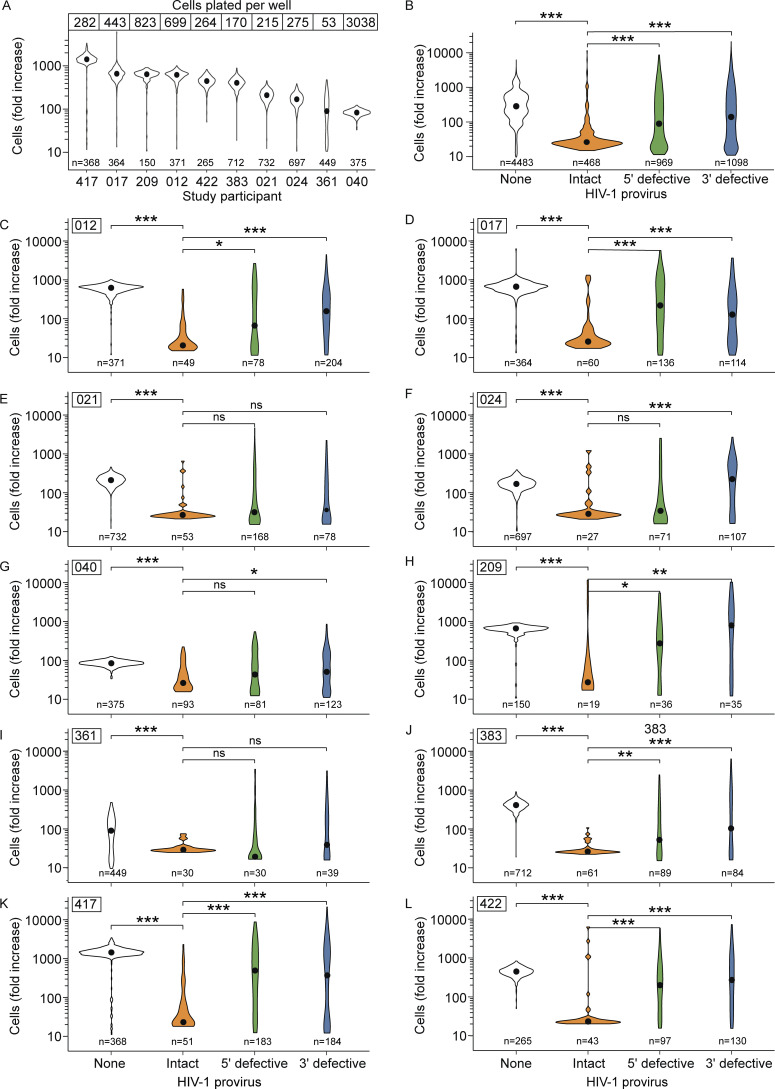
**Uninfected CD4**^**+**^ **T cells proliferate to a greater extent than infected CD4**^**+**^ **T cells following anti-CD3/anti-CD28 stimulation. (A)** Fold increase in total CD4^+^ T cells in individual culture wells from each study participant as measured by ddPCR analysis of the RPP30 gene. Black dots within each violin plot represent median values. **(B)** Average fold increase in uninfected CD4^+^ T cells or CD4^+^ T cells with the indicated type of provirus in individual culture wells following anti-CD3/CD28 stimulation. This is a pooled analysis from all 10 donors. **(C–L)** Fold change in CD4^+^ T cells stratified by HIV-1 provirus type for each study participant. Participant ID is displayed at the top of each panel. In this analysis, wells were not excluded if more than one provirus type was detected because the IPDA allows separate quantitation of the proliferation of each provirus type. Pairwise comparisons were performed with a Wilcoxon test. * P < 0.05, ** P < 0.01, *** P < 0.001, ns = not significant (P > 0.05).

In total, over 4,000 microcultures of resting memory CD4^+^ T cells from 10 study participants were analyzed. Total cell proliferation, reflecting predominantly uninfected cell proliferation, varied widely between individuals, ranging from an average increase in cell number of 83-fold for donor 040 to 1,433-fold for donor 417 (Fig. 3 A). These values correspond to an average of 6.5 and 10.5 divisions, respectively. Mathematical analysis of murine T cell responses to viral infection has shown that CD4^+^ T cells responding to an immunodominant epitope in lymphocytic choriomeningitis virus (LCMV) begin to divide between 48 and 72 h after stimulation and have a doubling time of 11 h in vivo (De Boer et al., 2003). Therefore, we expected to observe up to 12 divisions after 8 days. Variation in T cell proliferative responses between individuals has been observed, and T cell responsiveness declines with age (Vibert and Thomas-Vaslin, 2017; Ye et al., 2014). However, we did not observe a correlation between total cell proliferation and either study participant age, initial number of cells plated in each culture well prior to expansion, or culture time length (7 versus 8 days).

IPDA analysis showed that following a single TCR stimulation, memory CD4^+^ T cells with a genetically intact HIV-1 provirus generated far fewer progeny cells than did uninfected memory CD4^+^ T cells or memory CD4^+^ T cells carrying a defective provirus (Fig. 3 B). The number of progeny infected cells generated during these cultures reflects both the number of cell divisions and the death of infected cells. It is difficult to measure these parameters separately for infected cells which represent only a minute fraction of the CD4^+^ T cells present. However, culture conditions were optimized such that overall viability was >90% on day 7. The median fold increase for cells with an intact provirus was 25, representing between four and five divisions, assuming no cell death. The median fold increase of uninfected cells in the same culture wells was 284, or slightly more than eight divisions, assuming no cell death. The level of proliferation of infected cells with an intact provirus was significantly lower than that of uninfected cells for each study participant (P < 0.001) (Fig. 3, C–L). Interestingly, proliferation of cells with a defective HIV-1 provirus was intermediate between that of uninfected cells and cells with an intact HIV-1 provirus. Cells with a 5′ defective provirus (89-fold expansion) also proliferated less than cells with a 3′ defective provirus (112-fold expansion) (P = 0.003). While cells with intact proviruses consistently displayed low proliferation, cells with defective proviruses displayed more variable proliferation within and between study participants. This finding is consistent with the wide variety of defects arising from the deletions and hypermutation that can occur during reverse transcription (Bruner et al., 2019). Together, our results with memory CD4^+^ T cells from PWH on long-term ART show that HIV-1–infected cells, particularly those with an intact provirus, generate fewer progeny cells than uninfected cells following a single in vitro stimulation.

### Proliferation of cells with intact proviruses was reduced regardless of virion production

T cell activation can reverse HIV-1 latency, leading to productive infection and potential cell death through viral cytopathic effects. Therefore, to explain the reduced proliferation of infected CD4^+^ T cells, we first examined whether the production of virions restricted infected cell proliferation. Analysis of virion production is complicated by variation in burst size and the number of virions produced by a single infected cell. Estimates of the burst size range from 1,000 to 50,000 virions (Bui et al., 2015; Chen et al., 2007; De Boer et al., 2010; Hataye et al., 2019). Hataye et al. (2019) observed that there is also variation in the time at which virion production occurs following activation of latently infected cells. To ensure that we captured virions produced at different times during the culture, we froze aliquots of culture supernatant from each day of culture and pooled them for isolation of viral RNA (see Materials and methods). Combined supernatants were ultracentrifuged and viral RNA was isolated from the pelleted virus as previously described (Tosiano et al., 2019). The number of HIV-1 virions was then quantified using the VQA as previously described (Bullen et al., 2014; Shan et al., 2013). This strategy ensures that we can detect virion production, although we cannot establish when virion production occurred during the 7–8 days following stimulation.

**Figure S1.**
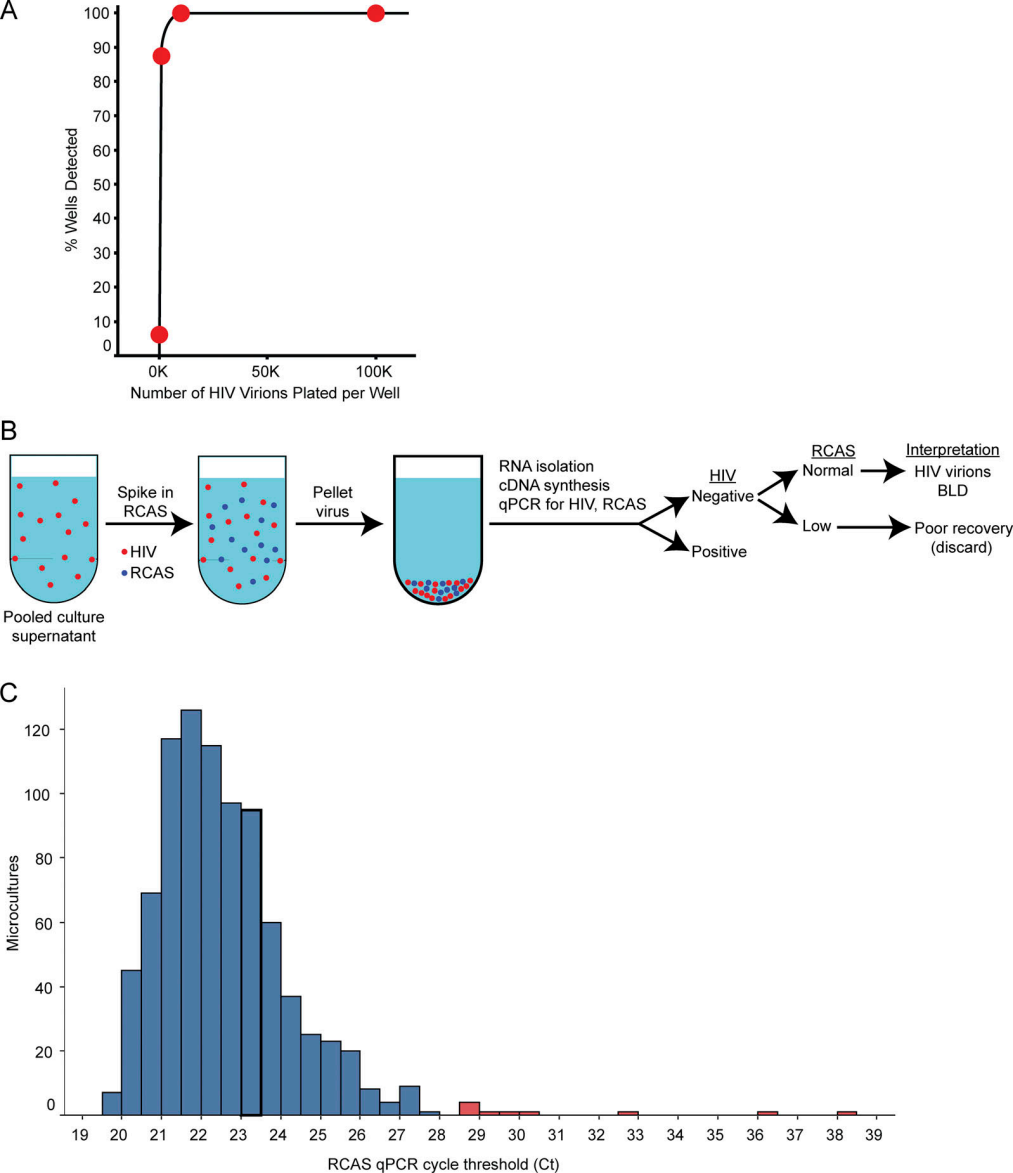
**Assay for virion production. (A)** Detection of HIV-1 virions in culture supernatants. To determine the sensitivity of the assay for supernatant virions, HIV-1 pseudoviruses were generated by transfection, pelleted by ultracentrifugation, and quantified using a p24 ELISA. The indicated number of pseudoviruses were subjected to one freeze/thaw cycle, added to a volume of culture media equal to that of the pooled supernatants harvested from a single culture well, and processed by the same steps of ultracentrifugation, RNA isolation, and quantification used for the culture supernatants (see Materials and methods). Graph shows the fraction of replicates with detectable HIV-1 RNA as a function of the input number of pseudoviruses. 16 experimental replicates were analyzed per condition. **(B)** Use of an internal standard to monitor recovery of viral RNA. To verify that wells negative for HIV-1 RNA had low or absent virion production, we measured the RCAS internal standard virus in each well (Palmer et al., 2003). BLD = below limit of detection. **(C)** Histogram displaying the distribution of RCAS internal standard virus qPCR cycle thresholds (Ct) after viral RNA extraction. Samples were considered to have normal recovery (blue) or poor recovery (red) based on visual inspection of the curve. Samples with poor RNA recovery were excluded from further analysis.

In validation experiments, we established that this assay could routinely detect 1,000 virions per pooled culture, a value that is at the lower end of the range of burst size measurements for individual HIV-1–infected cells (Fig. S1 A). To ensure that assay results below the limit of detection (LOD) represented low or absent virion production rather than technical issues with RNA isolation, cDNA synthesis, or PCR, an aliquot of an irrelevant retrovirus (RCAS, or replication-competent ASLV long terminal repeat with a splice acceptor) was spiked into each thawed, pooled supernatant prior to viral RNA isolation (see Materials and methods) (Palmer et al., 2003) (Fig. S1 B). If the amount of RCAS recovered was significantly low (see Materials and methods), then the sample was discarded (Fig. S1 C).

**Figure 4.**
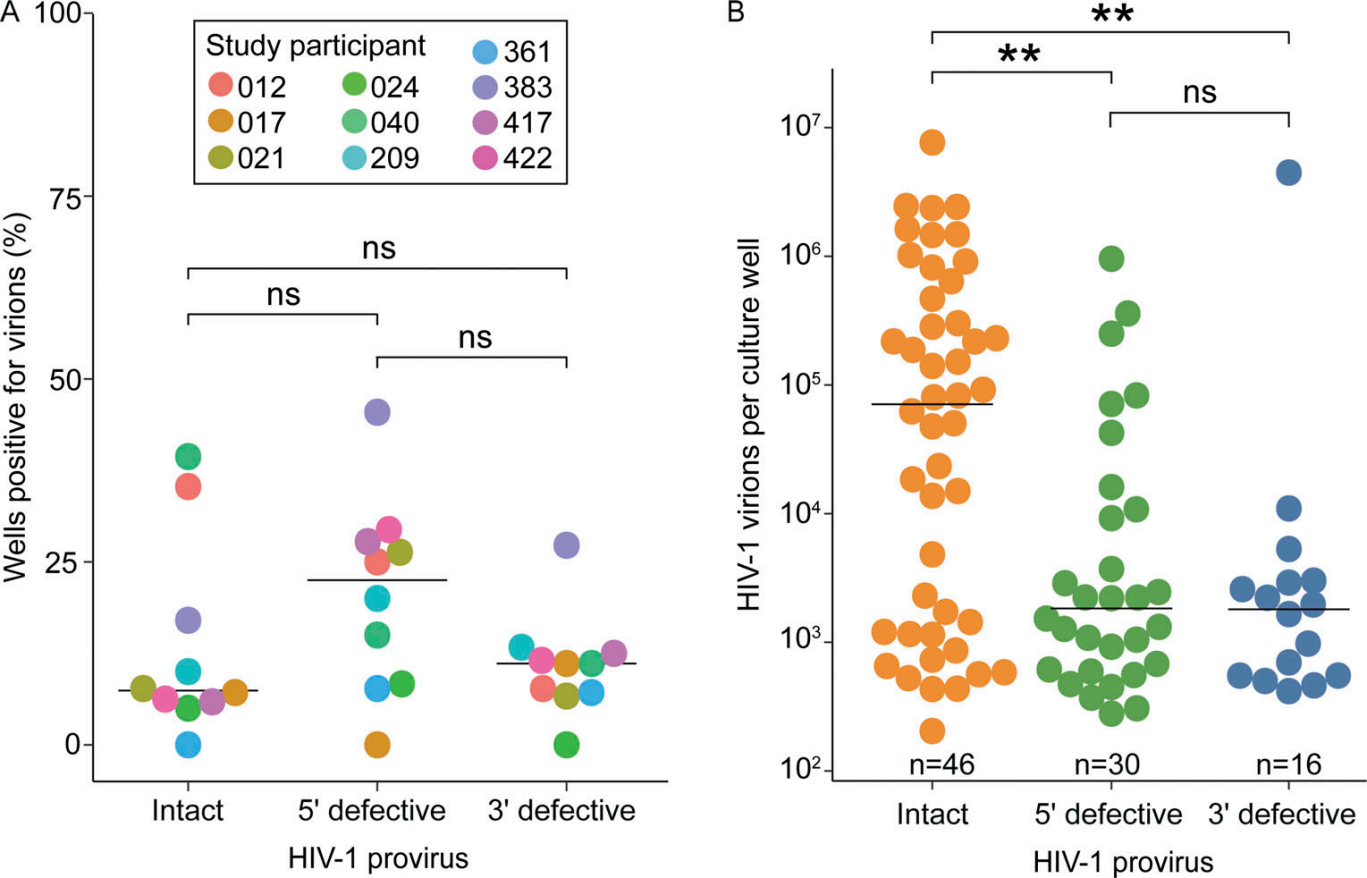
**Virion production by infected T cells. (A)** Fraction of cultures with detectable virion production for different types of HIV-1 proviruses. The percentage of wells with an intact, 5′ defective, or 3′ defective provirus that displayed detectable virion production is shown for each study participant (*n* = 563 total replicate wells). For this analysis, we have excluded wells with more than one provirus type detected by IPDA because some defective proviruses can give rise to virions, and we cannot be certain which provirus type is responsible for the virion production. **(B)** For positive wells, the number of virions per well produced from activated, infected CD4^+^ T cells harboring either an intact, 5′ defective, or 3′ defective provirus. Median values are shown for each provirus type (black lines). Only wells starting with a single provirus type were included in this analysis. Pairwise comparisons were performed with a Wilcoxon test. ** P < 0.01, ns = not significant (P > 0.05).

**Figure S2.**
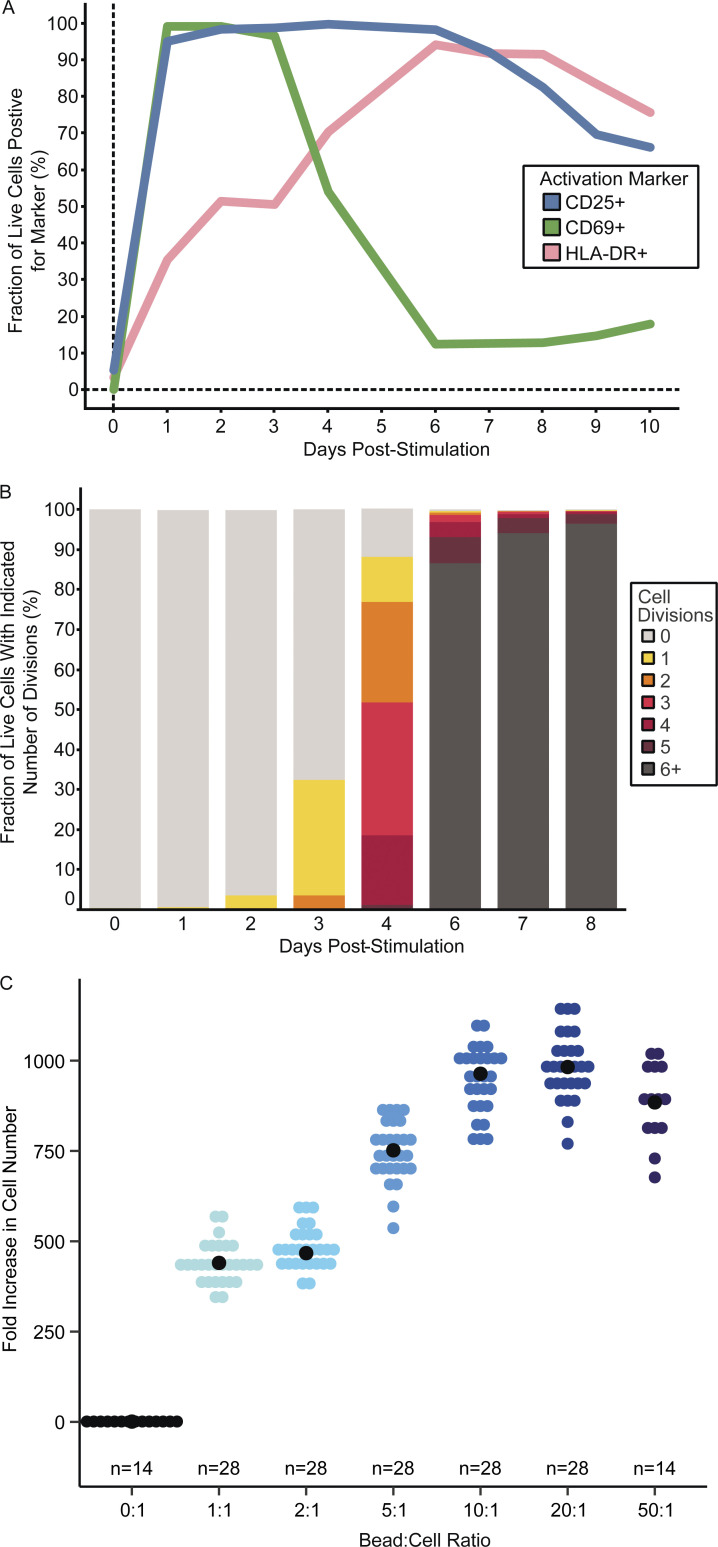
**Optimal CD4**^**+**^ **T cell stimulation conditions. (A and B)** (A) Activation markers and (B) number of cell divisions of memory CD4^+^ T cells from a PWH stimulated at high cell density on day 0 for 24 h prior to distribution into 96-well culture plates on day 1. A 1:1 anti-CD3/CD28 bead-to-cell ratio was used. **(C)** Fold increase in total healthy donor CD4^+^ T cells from a starting number of 800 cells per well after 7 days of stimulation with the indicated anti-CD3/CD28 bead:cell ratio. Each data point is a replicate microculture well. Median fold increase for each condition is represented by a black dot.

We detected virion production in only 46 of 278 wells (16.5%) with an intact HIV-1 provirus (Fig. 4 A). The percentage of wells that produced virions varied by study participant. For example, no wells with intact proviruses from participant 361 had detectable virion production, while over 25% of cultures with intact proviruses from participants 040 and 012 were positive for virion production. The absence of detectable virus production in most wells with an intact provirus was not due to a failure of T cell activation. As discussed above, cells with an intact provirus divided a minimum of about four to five times, yielding at least 16-fold expansion during the culture. In addition, the activation conditions used induced uniform activation of the input CD4^+^ T cells as assessed by expression of activation markers (Fig. S2 A) and cell proliferation (Fig. S2, B and C). Nevertheless, most cells carrying intact HIV-1 proviruses were not induced to produce virions by a single round of in vitro T cell activation. This finding is consistent with previous studies using the quantitative viral outgrowth assay (Ho et al., 2013; Hosmane et al., 2017; Kwon et al., 2020). Thus, proviral induction is not always directly coupled with T cell activation and proliferation.

Recent studies indicated that some cells with some types of defective proviruses can also be induced to express viral genes and in some cases produce virions (Imamichi et al., 2016; Pollack et al., 2017; White et al., 2023). We detected virion production in 16.1% (46/285) of wells containing a defective HIV-1 provirus. Among defective proviruses, 5′ defective proviruses were about twice as likely as 3′ defective proviruses to produce HIV-1 virions, possibly due to the absence of *tat* and *rev* in many 3′ defective proviruses (Bruner et al., 2019) (Fig. 4 A). Intact *gag* and RNA packaging signal (*psi*) are required for the production of virus particles carrying genomic viral RNA. Many 5′ defective proviruses have defects in the 5′ leader that do not prevent packaging of viral RNA (Bruner et al., 2019; Cole et al., 2021; Ho et al., 2013; Pollack et al., 2017; White et al., 2023). Conversely, most 3′ defective proviruses are defective in *tat*, *rev*, or *env* and may not be capable of the high-level production of Gag protein needed for virion production (see Discussion) (Bruner et al., 2019).

Although similar fractions of intact and defective proviruses produced virions, intact proviruses produced a much higher number of virions per well (Fig. 4 B). The median number of virions produced in a well containing a genetically intact provirus was nearly two logs higher than that of wells with either a 3′ defective or 5′ defective provirus. There was no difference between the quantity of virions produced in wells with a 5′ defective provirus and wells with a 3′ defective provirus (Fig. 4 B).

**Figure 5.**
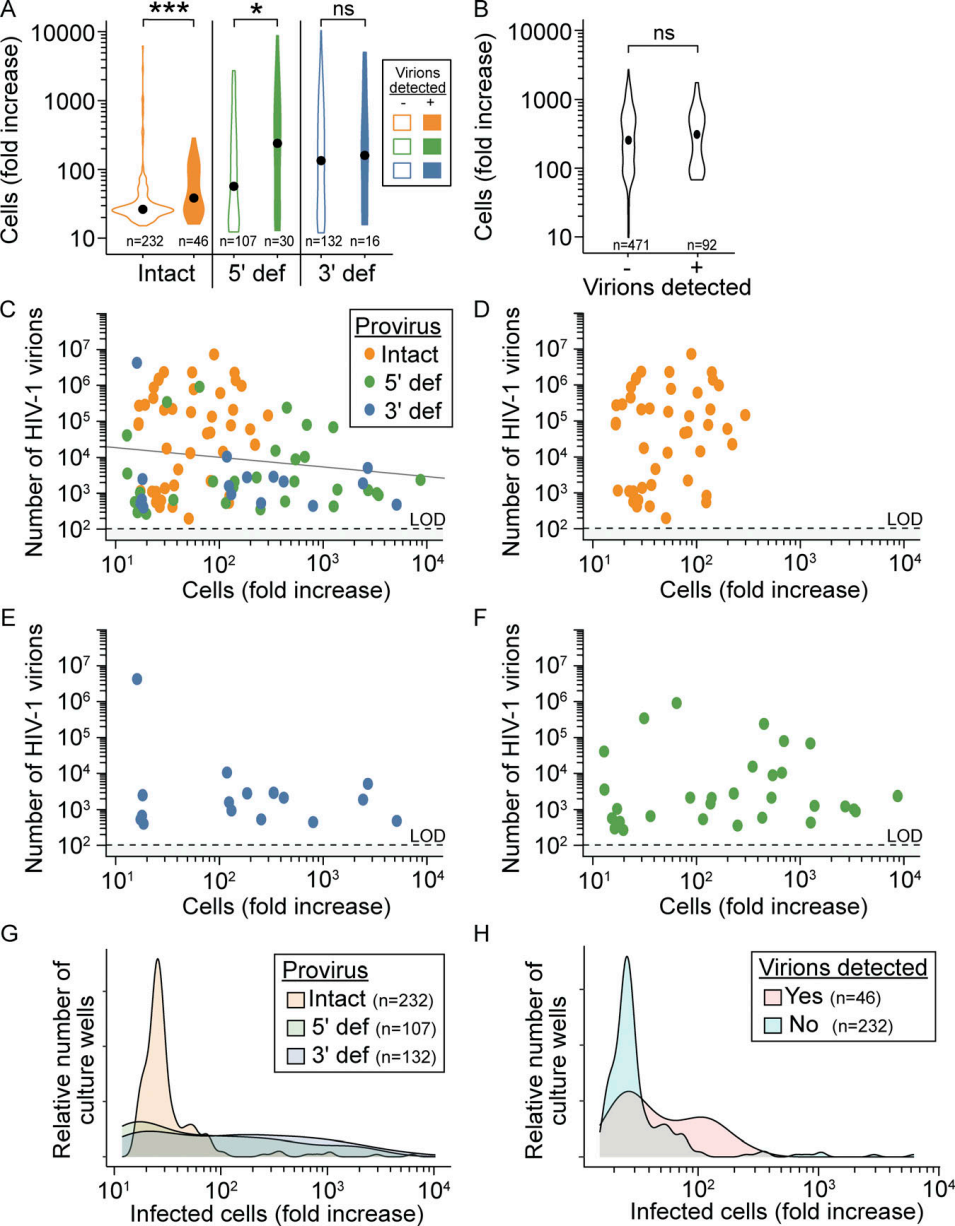
**Effect of virion production on proliferation of CD4**^**+**^ **T cells. (A)** Fold increase in cell number from single infected CD4^+^ T cells with either an intact or defective provirus following anti-CD3/CD28 stimulation. Black dots represent median values. For this analysis, we have excluded wells with more than one provirus type detected by IPDA because some defective proviruses can give rise to virions, and we cannot be certain which provirus type is responsible for the virion production. **(B)** Fold increase in uninfected cells in culture wells from A for which virus in the supernatant was detected or not detected. **(C–F)** Relationship between virion production and infected cell proliferation. For wells with detectable supernatant virions, the number of virions/well is plotted versus the fold increase in infected cells harboring (C) intact, 3′ defective, or 5′ defective proviruses by IPDA, (D) only intact proviruses, (E) only 3′ defective proviruses, or (F) only 5′ defective proviruses. Each dot represents a well starting with a single infected cell. **(G)** Density plot depicting infected cell proliferation in wells with no detectable supernatant virus. The relative number of culture wells with the indicated fold increase in infected cells is shown for wells with intact, 3′ defective, or 5′ defective proviruses by IPDA. **(H)** Density plot depicting infected cell proliferation in wells with an intact provirus by IPDA. The relative number of culture wells with the indicated fold increase in infected cells is shown for wells with and without detectable supernatant virus. Pairwise comparisons were performed with a Wilcoxon test. * P < 0.05, *** P < 0.001, ns = not significant (P > 0.05).

Most importantly, we asked if the production of virions restricted CD4^+^ T cell proliferation. Cells with intact proviruses produced the highest number of virions per well (Fig. 4 B) and generated the fewest progeny cells (Fig. 3 B). Due to the potential cytopathic effects of viral gene expression and the short half-lives of productively infected cells (Chen et al., 2002; Ho et al., 1995; Laurent-Crawford et al., 1993; Wei et al., 1995), we hypothesized that cells with an intact provirus would generate fewer progeny cells if high levels of virus were produced. Interestingly, the opposite was true: cells with intact proviruses displayed higher proliferation if there was detectable supernatant virus (median 38-fold expansion versus a 26-fold expansion in wells without detectable virus production, P < 0.001, Fig. 5 A). The same was true for proviruses with a 5′ defect. Wells with 3′ defective proviruses displayed the same level of proliferation whether virions were detected or not. Importantly, in wells with infected cells, the proliferation of uninfected cells was not affected by the presence or absence of supernatant virus (Fig. 5 B). This is the expected result given that antiretroviral drugs were included in the cultures to prevent de novo infection events.

We then looked more closely at the relationship between proliferation and virus production for wells with detectable supernatant virus. In cultures with detectable virions, there was no correlation between infected cell proliferation and the amount of virus produced (R^2^ = 0.02, P = 0.17) (Fig. 5 C). Individual plots for wells with intact, 3ʹ defective, and 5ʹ defective proviruses (Fig. 5, D–F, respectively) also revealed no significant relationship between proliferation and virus production.

The above results suggest that virus production is not strongly associated with reduced proliferation of infected cells, and it remains unclear why cells with an intact provirus generate fewer progeny than uninfected cells in response to TCR stimulation. To further examine the finding that virus production was not responsible for the reduced proliferation of cells with intact proviruses, we generated density plots depicting the level of infected cell proliferation for wells with or without detectable supernatant virus (Fig. 5, G and H). Even in the absence of detectable virus production, wells with intact proviruses displayed a peak of proliferation near the assay lower LOD (Fig. 5 G). In comparison, cells carrying defective proviruses displayed a flatter distribution in the level of cell proliferation. Nevertheless, the pronounced proliferative defect observed for cells with an intact provirus suggests that selective effects operating over long periods of time may give rise to infected cell populations with a relatively weak proliferative response that is not appreciably affected by virion production.

### Integration site analysis reveals an uncoupling between cell proliferation and latency reversal following TCR stimulation

To further investigate the relationship between CD4^+^ T cell proliferation and virion production, we used linker-mediated PCR to analyze the integration sites of intact and defective HIV-1 proviruses in 58 wells with different levels of virion production (see Materials and methods). By IPDA, 11 of these proviruses were intact, 24 had 5′ defects, and 23 had 3′ defects (Table S4). Two of the proviruses that gave double positive IPDA dot plots were shown by nFL sequencing to have a small number of G→A mutations in the sequence context typical of APOBEC3G-induced mutation (Table S4). Proviruses from 13 of the 58 wells had integration sites identical to sites found in other wells from the same donor, indicating enormous in vivo expansion of infected T cell clones. There were 45 wells with a unique site not seen in any other well analyzed. Among the 13 wells with sites observed more than once, there were a total of three distinct sites. Thus, a total of 48 different integration sites were identified in this analysis.

The 10 individuals in this study were on ART for a median of 17.5 years (range: 10–21). Consistent with previous studies of proviral integration sites in treated PWH, 92% of integration sites were within genes (17% exonic, 75% intronic) and 8% were intergenic (Cohn et al., 2015; Han et al., 2004; Ho et al., 2013; Ikeda et al., 2007; Mitchell et al., 2004; Schröder et al., 2002). For integration sites within genes, 64% were in the same orientation as the host gene and 36% were in the opposite orientation. For this analysis, HIV-1 integrations were only counted once, even if they were found in multiple wells. There was no significant enrichment for high proliferation or virion production based on intron/exon location or orientation. No integration sites were located in centromeric regions, and three out of the 48 distinct sites were located in genes belonging to the zinc finger (ZNF) family. Previous studies reported an enrichment of proviruses integrated in certain ZNF genes in vivo both in individuals on long-term ART and in elite controllers, rare PWH who control HIV-1 without ART (Huang et al., 2021; Jiang et al., 2020; Lian et al., 2023). Integration into ZNF genes may prevent HIV-1 gene expression because of the presence of transcriptionally repressive heterochromatin marks. This in turn could endow the cell with a survival advantage. We found a >3,000-fold increase in cell number, representing between 11.5 and 13 divisions, for two out of the three wells with ZNF integration sites. Other infected cells displaying >3,000-fold increases in cell number had proviruses integrated in *CLUAP1*, *CARMIL1*, *XAF1*, *VPS13B*, and *TCERG1*. No integration sites were encountered in the small set of genes shown by Coffin et al. (2021) to provide a selective advantage in vivo. Overall, effects related to the site of integration alone were not related in any simple way to the in vitro proliferation observed in our study.

### In vivo clone size is negatively correlated with in vitro expansion

To better understand the proliferative potential of infected cells in vivo, we designed ddPCR primer and probe sets across the host–viral junctions (HVJs) of 11 proviruses for which we had matched in vitro proliferation, in vitro virion production, and integration site data. We then quantified the in vivo frequency of cells belonging to these clones among resting memory CD4^+^ T cells from the relevant donors. This strategy has been used previously to track individual proviruses in vivo (Anderson et al., 2020; Brandt et al., 2021; Simonetti et al., 2021).

**Figure 6.**
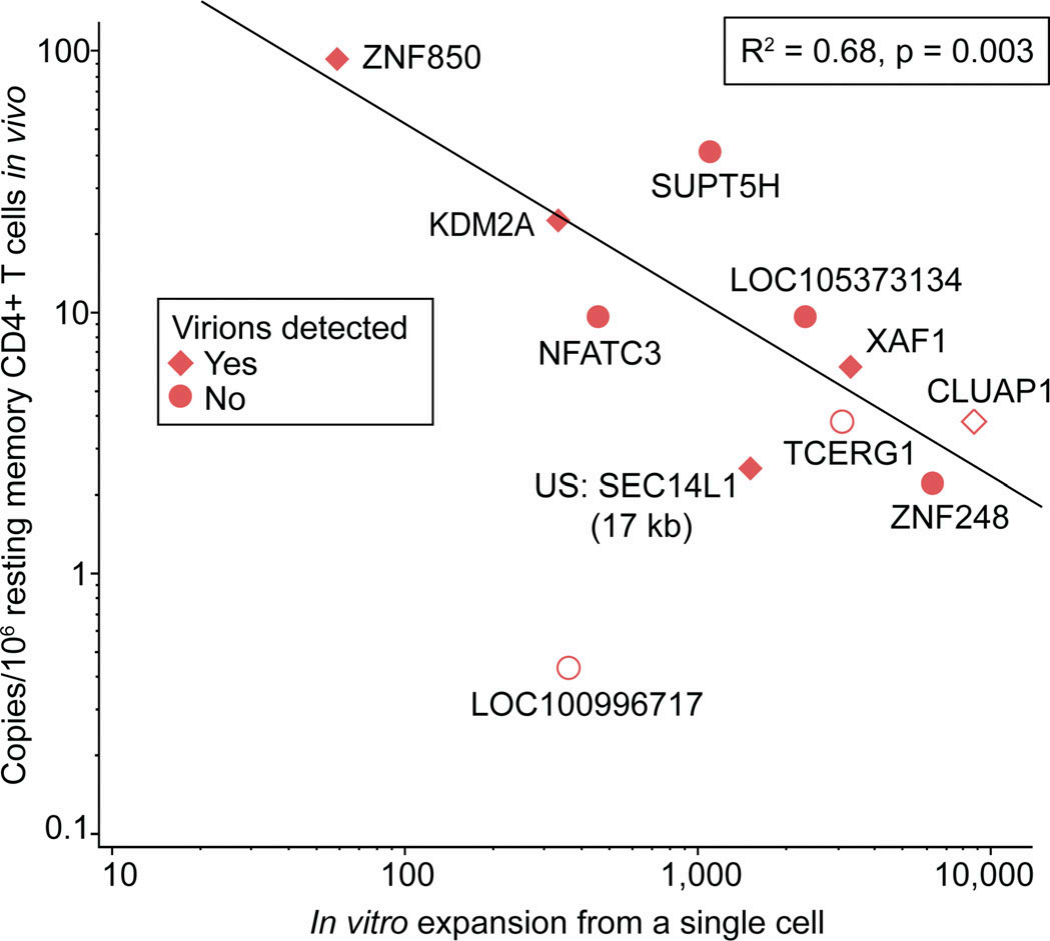
**Relationship between in vivo clone size and in vitro proliferation.** The in vivo frequency of each of the indicated clones of infected CD4^+^ T cells compared with its in vitro expansion starting from a single cell. Mean in vitro expansion is plotted for clones encountered in multiple microculture wells (see Table S5). Each data point represents an infected cell clone labeled with its proviral integration site. Symbol shape denotes the detection of HIV-1 virions in vitro. Empty symbols represent clones not detected in vivo and are plotted at 50% of the LOD, which was unique to each clone and proportional to the number of cell equivalents screened (see Table S5 and Materials and methods). Note that outlier LOC100996717 was excluded from the statistical analysis. US indicates the integration site was upstream of the indicated gene by the indicated distance.

Three of the proviruses were present in vivo at levels below the LOD of the ddPCR assay but nevertheless proliferated in vitro in the microculture assay (Table S5). The remaining eight proviruses were present at frequencies ranging from 2 to 94 copies per million resting memory CD4^+^ T cells. Interestingly, there was a significant negative correlation between the frequency of the infected cell clone in vivo and the amount of in vitro proliferation measured starting with a single infected cell from that clone (R^2^ = 0.68, P = 0.003) (Fig. 6). If the three proviruses not detected in vivo are excluded from the analysis, the correlation remains significant (R^2^ = 0.65, P = 0.02). In addition, there was no apparent relationship between in vivo clone size and in vitro virion production. These results further highlight the complexity of the proliferative responses that prevent reservoir decay.

## Discussion

A better understanding of factors underlying the remarkable stability of the latent reservoir will facilitate the development of effective curative strategies. The stability of the latent reservoir is due in part to the proliferation of latently infected memory CD4^+^ T cells (Bailey et al., 2006; Bui et al., 2017; Crooks et al., 2015; Hosmane et al., 2017; Lorenzi et al., 2016; Maldarelli et al., 2014; Simonetti et al., 2016; Tobin et al., 2005; Wagner et al., 2014). Using single-cell microcultures of CD4^+^ T cells from treated PWH, we have shown that there is a difference in the proliferation of infected and uninfected T cells after optimal CD3/CD28 stimulation. CD4^+^ T cells harboring a provirus classified as intact by the IPDA generated fewer progeny cells in these experiments than uninfected CD4^+^ T cells. This was true for every study participant. Cells carrying defective proviruses showed proliferative responses that were better than those of cells with intact proviruses but inferior to those of uninfected cells. To understand the mechanism underlying the reduced proliferation of cells with intact proviruses, we examined virus production following TCR stimulation. Although TCR stimulation induced some degree of proliferation in every well, virion production was detected in only a small fraction of cultures seeded with a cell carrying an intact provirus. Virion production was also detected in a similar fraction of wells with defective proviruses but at a much lower level. Surprisingly, for wells with intact proviruses, detectable virion production was not associated with reduced infected cell proliferation. In vivo clone size was inversely related to the in vitro proliferative response for individual clones. Together, these results shed light on the complex dynamics of the infected cell proliferation that prevents reservoir decay.

The in vitro experiments described here suggest that infected cells with a replication-competent HIV-1 provirus will likely generate fewer progeny cells following in vivo activation than would uninfected cells. One immediate clinical implication of this work is related to the use of non-specific anti-proliferative agents to block clonal expansion of infected cells (Schiffer et al., 2022). Our results indicate that these strategies may disproportionately affect the normal T cell response, resulting in greater toxicity than therapeutic benefit. Our findings also have important implications for understanding reservoir decay dynamics. Several groups have shown that over the first 7 years on ART, intact proviruses decay more rapidly than defective proviruses (Antar et al., 2020; Bruner et al., 2019; Cho et al., 2022; Fray et al., 2023; Gandhi et al., 2021; Peluso et al., 2020; White et al., 2022). However, we have recently shown that after the first several years of ART, the decay of cells with inducible, replication-competent provirus is reversed, and the frequency of these cells begins to slowly increase, likely due to infected cell proliferation (McMyn et al., 2023). In addition, several other studies have shown that large populations of cells carrying identical, replication-competent proviruses are present in PWH on ART (Bui et al., 2017; Hosmane et al., 2017; Lorenzi et al., 2016; Simonetti et al., 2016). Importantly, several studies have shown that the in vivo proliferation of infected cells is largely driven by the normal response to frequently encountered antigens (Collora et al., 2022; Mendoza et al., 2020; Simonetti et al., 2021). Thus, the reduced proliferative response described in our in vitro studies must be reconciled with the large amount of experimental data demonstrating extensive in vivo proliferation of infected cells.

The simplest explanation for the reduced proliferation of cells with an intact provirus is that T cell activation not only drives cell division but can also induce proviral reactivation and the production of viral proteins and virions. Most productively infected cells have a very short in vivo half-life (<1 day) (Ho et al., 1995; Wei et al., 1995). In addition, the HIV-1 Vpr protein induces cell cycle arrest by depleting a host protein whose function is required for progression through the G2/M phase (He et al., 1995; Jowett et al., 1995; Zhang and Bieniasz, 2020). Further studies have demonstrated that Vpr has highly pleiotropic effects on other host cell factors, suggesting it may perturb cell function well beyond cycle arrest (Jacotot et al., 2000; Qiao and McMillan, 2007; Schröfelbauer et al., 2007; Stewart et al., 1999). In addition, expression of other HIV-1 proteins including Env and Tat has been associated with cytopathic effects (Chen et al., 2002; Laurent-Crawford et al., 1993). For these reasons, we hypothesized that high levels of virion production would be associated with reduced proliferation. However, analysis of virus production in individual microculture wells revealed no simple relationship between virion production and proliferation. Specifically, for wells seeded with an intact provirus, no virus production was detected in most wells, and the level of infected cell proliferation was similar to that observed in wells with detectable virus production. These findings are discussed separately below.

Greater than 80% of wells seeded with an intact provirus were negative for detectable virion production despite robust total T cell proliferation in every well. This finding is consistent with previous studies showing that in limiting dilution viral outgrowth assays, some wells that are negative for outgrowth following the initial stimulation become positive after subsequent stimulations (Ho et al., 2013; Hosmane et al., 2017; Kwon et al., 2020). The apparently stochastic nature of proviral induction despite optimal T cell activation could reflect epigenetic silencing, transcriptional interference, stochastic processes involving the Tat-dependent positive feedback loop, proviral induction without virion production, or effects related to the site of integration (Hataye et al., 2019; Mbonye and Karn, 2017; Pearson et al., 2008; Shan et al., 2011; Weinberger et al., 2005). The apparently stochastic nature of proviral induction could provide one explanation for the generation of large clones of infected cells in vivo. Cells carrying replication-competent proviruses could be stimulated by antigen to proliferate, and, if the provirus is not induced, the cells could avoid viral cytopathic effects and immune clearance.

The reduced proliferation we observed in vitro was not clearly related to virion production. Cells with an intact provirus generated fewer progeny than uninfected cells, regardless of whether or not virions were detected in the supernatant. One intriguing possibility is related to the necessity of controlling T cell proliferation in vivo (Chamoto et al., 2023). Clones that are specific for frequently encountered antigens or that proliferate robustly following antigen exposure may be more susceptible to the negative control mechanisms that keep T cell proliferation in check and enforce contraction phase elimination of recently activated T cells. As the clones expand over long periods of time, the cells could become progressively less capable of further clonal expansion. The probability of initial infection and subsequent antigen-driven activation are both dependent on the frequency of antigen exposure. Several recent studies have identified infected CD4^+^ T cell clones specific for commonly encountered antigens such as CMV and HIV-1 (Collora et al., 2022; Mendoza et al., 2020; Simonetti et al., 2021). These clones must have expanded from cells infected before the initiation of ART (>10 years before sampling in our study). Recent studies have demonstrated that large in vivo clonal populations of HIV-1–infected cells are enriched in the effector memory subset with a lower proliferative potential, likely due to chronic antigen exposure (Anderson et al., 2020; Cole et al., 2022; Duette et al., 2022; Gantner et al., 2020; Simonetti et al., 2021). In contrast, the uninfected memory CD4^+^ T cell population could include cells generated more recently and cells specific for less commonly encountered antigens, cells that would be less affected by the processes described above. In addition, recent phenotypic studies have demonstrated upregulation of inhibitory checkpoint markers such as PD-1 and CTLA-4 on HIV-1–infected cells that could inhibit their proliferation (Fisher et al., 2023; Pardons et al., 2019; Neidleman et al., 2020; Rasmussen et al., 2022). For these reasons, an extensive history of prior in vivo clonal expansion occurring over many years could result in populations of infected cells with reduced residual proliferative potential. In this way, evidence for in vivo clonal expansion can be readily reconciled with the reduced in vitro proliferation observed here. This model is supported by our observation that cells carrying intact proviruses show reduced in vitro proliferation relative to uninfected cells regardless of whether virions are detected in the culture. The negative correlation that we observed between in vivo clone size and in vitro expansion also points to in vivo mechanisms operating over time that influence infected cell proliferative potential, such as the memory subset harboring the infected cell clone.

The model described above does not fully account for the behavior of cells with defective proviruses, which displayed a much wider range of proliferative responses (Fig. 3, B–L). This finding is consistent with the wide variety of defects present in these proviruses. Each defective provirus has a unique biology that depends on the precise location and size of the deletion and/or the pattern of hypermutation. Some defective proviruses have defects in every ORF and would likely be incapable of expressing viral genes (Bruner et al., 2019). Cells carrying these proviruses may behave like uninfected cells. Other proviruses with less severe effects are capable of viral gene expression, and depending on the nature of the defect, virion production. Interestingly, we found that a similar percentage of wells with a defective HIV-1 provirus produced virions compared with those with an intact provirus. However, the level of virus produced was much lower (Fig. 4 B). Virus production from defective proviruses has been observed previously (Fisher et al., 2022; Imamichi et al., 2016; Pollack et al., 2017; White et al., 2023). As long as the Gag protein is produced, a cell carrying an HIV-1 provirus can release virus-like particles (Gheysen et al., 1989).

The wide range of proliferative responses of cells with defective proviruses indicates that proliferative potential is not simply a function of the age of the memory cell clones as marked by the time on ART, given that the same temporal parameter would apply to all infected cells. Rather, there must be a component of this phenomenon that depends on the nature of the provirus.

In conclusion, we have demonstrated at the single-cell level that latently infected CD4^+^ T cells with an intact HIV-1 provirus have a reduced proliferative response to TCR stimulation. The reduced response is independent of host immune pressure and is not directly related to measurable virion production. Of immediate clinical significance, this result provides evidence against the use of antiproliferative agents, suggesting that they may affect the normal T cell response more than they limit infected T cell proliferation. Mechanistically, our experiments suggest that the fate of an individual infected T cell may be influenced by multiple viral and cellular factors. Although our results do not directly address the in vivo proliferative potential of infected cells, we propose that the reduced in vitro proliferative response of infected T cells with an intact provirus may be explained by a low proliferation phenotype resulting from long-term in vivo clonal expansion.

## Materials and methods

### Study participant characteristics

Characteristics of the study participants are provided in Table S1. Participants were on suppressive ART for >10 years with undetectable plasma HIV-1 RNA levels for >9.7 years. Leukapheresis was performed for study participants 012, 017, and 040. For all other participants, a 150 ml peripheral blood sample was collected.

### Isolation of resting memory CD4^+^ T cells

Total peripheral blood mononuclear cells (PBMCs) were isolated by density centrifugation using Ficoll-Paque PLUS (GE Healthcare Life Sciences). Total CD4^+^ T cells were then enriched from PBMCs using negative immunomagnetic selection with the EasySep Human CD4^+^ T Cell Enrichment Kit (StemCell Technologies). Resting memory CD4^+^ T cells (CD4^+^, CD69^−^, CD25^−^, HLA-DR^−^, and CD45RA^−^) were isolated using a second negative selection step (CD25-Biotin; Anti-Biotin MicroBeads; CD69 MicroBead Kit II; Anti–HLA-DR MicroBeads, Anti-CD45RA Microbeads; all from Miltenyi Biotec). Memory CD4^+^ T cell purity was >99% as assessed using flow cytometry specific for the CD4^+^, CD45RO^+^ population.

### Reservoir measurement

Genomic DNA (gDNA) was extracted from an aliquot of resting memory CD4^+^ T cells using the Qiagen DNA Mini kit. IPDA was performed on gDNA as previously described to quantify genetically intact, 3′ defective, and 5′ defective HIV-1 proviruses (Bruner et al., 2019). ddPCR for RPP30 was performed to measure total cell equivalents per reaction and correct for gDNA shearing. The results from the IPDA and RPP30 assays were assessed together to calculate the frequency of intact, 3′ defective, and 5′ defective proviruses per million resting memory CD4^+^ T cells as previously described (Bruner et al., 2019).

### Cell stimulation, plating scheme, and culture

Concurrently with the reservoir measurement above, an aliquot of resting memory CD4^+^ T cells was pelleted and resuspended in stimulation media. This media consisted of RPMI containing 10% fetal bovine serum (FBS), 100 U/ml of IL-2, 10 μM FTC, 10 μM TDF, and 10 million anti-CD3/CD28 Dynabeads/ml, resulting in a 10:1 Dynabead:T cell ratio. Cells were plated in 12-well culture plates, with 1.75 million cells/well in 1.75 ml of stimulation media, resulting in a concentration of 1 million cells/ml and a surface density of 1 million cells/2 cm^2^. These conditions were chosen to ensure maximum T cell activation and proliferation. Activation was assessed by expression of CD69, CD25, and HLA-DR, each of which shows a different characteristic time course of upregulation following TCR stimulation (Fig. S2 A). Cell division was quantified using dilution of carboxyfluorescein diacetate succinimidyl ester (Fig. S2 B). For the initial stimulation, we determined the optimal bead-to-cell ratio (10:1) which maximized T cell proliferation (946-fold expansion by day 7; Fig. S2 C). At this ratio, >98% of T cells were activated as assessed by CD69 expression at 24 h, and viability remained high at day 7.

After 24 h, the cells were distributed into 96-well plates in two plating schemes (Table S2). Based on the initial IPDA measurement, the number of CD4^+^ T cells plated per well was such that each well would contain on average one infected cell (plating scheme 1) or an average of 0.35 infected cells, representing limit dilution (plating scheme 2). Immediately prior to plating, the concentration of cells was measured directly to account for any loss during the 24-h stimulation. The cells were then diluted appropriately in RPMI containing 10% FBS, 100 U/ml of IL-2, 10 μM FTC, and 10 μM TDF and plated in a 96-well format according to the plating scheme, for a total volume of 250 μl per well.

Beginning 48 h after stimulation, a media change was performed every 24 h until the end of the culture. 200 μl of media was removed from each well without disturbing the cultures. The media removed from each well was frozen and reserved for HIV-1 virion analysis. 200 μl of fresh media was then added to the cultures.

Most cultures proceeded until day 8 after stimulation, but the cultures for study participants 012 and 040 were halted at day 7 as they had already reached maximal cell density. At the end of the cultures, the cells were pelleted and all media was removed and frozen. The cells were resuspended in 60 μl of PBS, transferred to a 96-well deep-well culture plate (NUNC), and frozen.

### Extraction of gDNA

96-well deep-well culture plates containing frozen culture cells were thawed, and gDNA was isolated using the Zymo 96 DNA kit according to the manufacturer’s instructions with several adjustments. The ratio of lysis buffer to cell suspension used was 6:1. Immediately upon adding lysis buffer, the mixture was vortexed using a plate vortexer for 15 s (3,000–3,500 RPM). To optimize the elution of purified gDNA, 30 μl of elution buffer was used, and the elution step was repeated twice by adding the eluant back to the column (for a total of three elution steps). This optimized protocol significantly lessened PCR inhibition and increased gDNA concentration at the expense of overall DNA yield. The concentrated DNA could then be used directly for IPDA analysis.

### IPDA analysis of infected cell proliferation

We used the IPDA to quantitate infected cell proliferation because the short, highly efficient PCRs involved allow quantitative detection of target sequences (Bruner et al., 2019). Any loss of HIV-1 proviruses would therefore result from DNA loss during isolation or from shearing. Shearing is corrected by using ddPCR for the host gene RPP30 (Bruner et al., 2019). Based on the fraction of the isolated gDNA analyzed and the level of DNA shearing, we estimated that we could detect proviruses if individual infected cells underwent four to five divisions during culture. We tested this estimate by sorting the exact numbers of immortalized infected T cells (J-Lat clone 10.6, RRID:CVCL_8281) (Jordan et al., 2003) into replicate culture wells containing 350,000 healthy donor CD4^+^ T cells. gDNA was immediately extracted, and 36% of the gDNA was analyzed using the IPDA. In line with the above calculation, the optimized gDNA recovery method resulted in the detection of 20 infected cells with a 50% detection rate and 60 infected cells with a 90% detection rate. The IPDA was performed as previously described (Bruner et al., 2019). Briefly, 8 μl of gDNA from each well was added to a mastermix consisting of ddPCR supermix (no deoxyuridine triphosphates), IPDA primers and probes, and diethyl pyrocarbonate–treated water, for a total reaction volume of 22 μl per well. In parallel, gDNA from uninfected donor CD4^+^ T cells and J-Lat 10.6 cells were run as negative and positive controls, respectively, on each sample plate.

### Provirus type determination

2D plots of the fluorescent signals from IPDA droplets were analyzed and interpreted for each culture well as previously described (Bruner et al., 2019). Wells were considered negative for infected cells if there were no fluorescent droplets. Wells showing positive droplets only in the 5(6)-carboxyfluorescein (FAM) channel were considered to contain a 3′ defective provirus. Wells with positive droplets only in the 2′-chloro-7′-phenyl-1,4-dichloro-6-carboxyfluorescein (VIC) channel were considered to contain a 5′ defective provirus. Wells with both types of single-positive droplets and no double-positive droplets were considered to have been seeded with two infected cells, one harboring a 3′ defective provirus and one harboring a 5′ defective provirus. Wells containing individual droplets positive in both the FAM and VIC channels were considered to have been seeded with an infected cell with an intact HIV provirus. For wells containing intact proviruses, some single positive droplets are expected: DNA shearing during gDNA extraction causes about 30% of intact proviruses to be sheared in between the two amplicons, resulting in one droplet positive in FAM and one droplet positive in VIC per provirus (Bruner et al., 2019). ddPCR for RPP30 performed for each well was used to correct for shearing. Wells were considered to have only an intact provirus if the raw number of double positive droplets exceeded the average number of raw single positive droplets in each channel. This reflects normal DNA shearing. However, in wells with evidence of an intact provirus, the presence of an additional 3′ defective or 5′ defective provirus was inferred if the number of droplets positive in a single channel was more than twice as high as the number of droplets positive only in the other channel.

### Quantification of cell proliferation

Infected cell proliferation was quantified using IPDA measurement of the number of HIV-1 proviral copies in the gDNA from a given microculture well (Bruner et al., 2019). With the assumption that each well started with no more than one infected cell, this was a direct measure of infected cell proliferation. Uninfected cell proliferation was measured by performing RPP30 on gDNA from each culture well. The calculation of uninfected cell fold change was performed using the following formula:

$$Uninfected\;cell\;proliferation=\frac{\left(\#\;total\;cells\;by\;RPP30\right)-\left(\#\;infected\;cells\;by\;IPDA\right)}{\left(Avg.\;\#\;cells\;plated\;per\;well-1\right)}.$$

RPP30 was also used as a shearing control for IPDA, as previously described (Bruner et al., 2019). Rare wells with a total cell fold increase of 10 or less were discarded due to poor overall proliferation.

### RCAS internal virion standard

High-titer RCAS stocks were obtained by transfecting UMNSAH/DF-1 cells (Cat# 0307, RRID:CVCL_0570; BCRJ) with the RCASBP(A)gfp plasmid (a gift of S. Hughes, National Cancer Institute, Bethesda, MD, USA) and harvesting the supernatant on days 7 and 10 after transfection (Palmer et al., 2003). RCAS stocks were diluted in tris-buffered saline and frozen. Stocks were thawed, diluted further, and measured by real-time RT-PCR assay. In preparation for use as an internal standard, RCAS stock was thawed and diluted in tris-buffered saline immediately before use. 10 μl of the diluted RCAS was added to each pooled microculture media sample as an internal control for virion recovery.

### Extraction of viral RNA in cell culture supernatant

Frozen cell culture supernatants from days 2–8 were thawed for 15 min at 25°C and then combined in microcentrifuge tubes. To each tube, 10 μl of RCAS was added as an internal standard (Palmer et al., 2003). Then, RNA was isolated as previously described (Tosiano et al., 2019).

### Synthesis of standards for HIV-1 RNA and RCAS RNA quantification

RNA standards for the VQA and RCAS qPCR assays were generated as follows. Using as a template a previously described plasmid containing the HIV-1 VQA amplicon, we added a T7 promoter region (5′-TAA​TAC​GAC​TCA​CTA​TAG​GGA​GA-3′) to the 5′ end of the product, with forward primer 5′-TAA​TAC​GAC​TCA​CTA​TAG​GGA​GAC​TTG​TTA​CAC​CCT​GTG​AGC​CTG-3′ and reverse primer 5′-TTT​TTT​TTT​TTT​TTT​TTT​TTT​TTT​TTT​TTT​TTT​GAA​GCA​CTC​AAG​GC-3′ (Bullen et al., 2014). Similarly, using the RCASBP(A)gfp plasmid as a template, we added the same T7 promoter sequences to the 5′ end of a region of RSV *gag* with forward primer 5′-TAA​TAC​GAC​TCA​CTA​TAG​GGA​GAC​ATT​GAC​TGC​TTT​AGG​CAG​AAG​TCA​C-3′ and reverse primer 5′-AAC​AGC​GCG​GTG​ATA​TAC​ACC-3′ (Palmer et al., 2003). A Dpn1 digest then degraded any remaining plasmid template. Dpn1 was removed using the Clontech PCR Clean-up kit. The resulting VQA and RCAS PCR products were transcribed using the MEGAscript T7 kit (Ambion), including a DNase step at the end to degrade the DNA template. The resulting RNA was confirmed to be of the correct size before PCR cleanup with the RNeasy kit (Qiagen). RNA products were quantified spectrophotometrically at 260 nm and stored at −80°C in single-use aliquots of standard stocks at 10^9^ copies/μl. These aliquots were thawed and further diluted to assemble standard curves for VQA and RCAS RT-PCR assays.

### Quantification of HIV-1 virions in cell culture supernatants

In vitro–transcribed VQA and RCAS RNA standards were serially diluted in parallel to generate standard curves. RNA standards and freshly isolated experimental viral RNA samples were measured simultaneously as follows. RNA was converted into cDNA using qScript cDNA Supermix (QuantaBio) according to the manufacturer’s instructions. cDNA was then quantified in triplicate using the VQA qPCR assay as previously described to measure HIV-1 RNA copies (Bullen et al., 2014; Shan et al., 2013). In parallel, RCAS was measured using a previously described RCAS qPCR assay to estimate total RNA recovery (Palmer et al., 2003). Standard cDNA was run in duplicate on each VQA and RCAS sample qPCR plate. Averaged standard curves representing all analysis runs were used for downstream analysis (below) to control for Poisson variability in diluted standard RNA aliquots derived from the same stock.

To quantify HIV-1 RNA copies using VQA, triplicate experimental Ct values were first averaged. In rare cases when an experimental sample yielded some detectable and some undetectable replicates, the maximum possible Ct value (45) was assigned to replicates with undetectable viral RNA to enable the calculation of a quantitative value for viral RNA. Using the average VQA Ct value, viral RNA quantities were then extrapolated from the averaged VQA standard curve. The number of HIV-1 viral RNA copies was converted to HIV-1 virions, assuming two HIV-1 mRNA genomes per virion and using an experimentally determined viral RNA recovery. This recovery value was determined by plating known quantities of pseudotyped HIV-1 virions before immediately extracting and quantifying viral RNA.

For RCAS, cDNA was diluted prior to quantification by qPCR. The experimental Ct was dilution-corrected by extrapolating RNA copies from the averaged RCAS standard curve, multiplying the RNA copies by the dilution factor, and using the same averaged RCAS standard curve to extrapolate the dilution-corrected Ct. The RCAS Ct was used to determine whether viral RNA recovery was normal or poor. Experimental samples were discarded in the case of poor RNA recovery, which occurred if the RCAS Ct value was an outlier, defined as significantly high by visual inspection of the curve (Fig. S1 C). Visual inspection was used instead of Z-score to determine outlier status because the distribution of Ct values could not be statistically confirmed as normal (P > 0.05).

### Whole genome amplification (WGA) of gDNA

gDNA from selected microculture wells was distributed into 96-well PCR plates and subjected to WGA by multiple displacement amplification using the non-advanced or advanced Single Cell Repli-G Kit (Qiagen), following the manufacturer’s protocol. Whole genome-amplified samples were diluted 25-fold in 10 mM Tris HCl, pH 8.0. Diluted samples were quantified by Qubit 3 Broad Range (Thermo Fisher Scientific), and HIV-1 amplification was assessed using IPDA. gDNA from successful WGA reactions was used as a template for integration site analysis and/or HIV-1 nFL proviral sequencing.

### Integration site analysis

Integration site analysis was done using the Lenti-X Integration Site Analysis Kit (Takara Bio) according to the manufacturer’s protocol. All 10 study participants were represented in this analysis and samples were only assayed if IPDA analysis revealed the presence of a single provirus type. Amplification of both the 5′ and the 3′ HVJs was performed using a primer specific for a universal adapter and another primer in either the HIV-1 5′ LTR or 3′ LTR. Putative integration sites were considered valid with high confidence if more than one restriction digest yielded the same integration site, if both 5′ LTR and 3′ LTR amplifications yielded the same site, or if the same site was identified in more than one microculture sample from the same study participant.

### nFL proviral sequencing

To verify proviral intactness for samples with recovered integration sites, whole-genome amplified gDNA from microculture wells was subjected to five overlapping nested PCR amplicons amplifications (∼2,000 nt each) as previously described to provide coverage of ∼9,000 nt of the HIV-1 genome (Einkauf et al., 2019; Lee et al., 2017). If amplification of all five regions was successful as indicated by the presence of gel bands of the correct size, the five PCR products were combined into a single tube and the sample was subjected to library preparation and next-generation sequencing (NGS). For one sample, nFL sequencing was achieved by Sanger sequencing the entirety of each of the five amplicons.

### Bioinformatic analysis of HIV-1 sequences

NGS sequence data (Johns Hopkins Medical Institute sequencing core) for each sample underwent de novo assembly to create a nFL contig using a previously established pipeline (Hiener et al., 2017). For the single sample that underwent nFL Sanger sequencing, the Sanger reads were assembled manually into a nFL contig. Sanger sequencing was also used to troubleshoot regions that were missing from incomplete contigs until nFL contigs were assembled for each sample. To assess proviral intactness, nFL contigs were aligned to HXB2 to check for the presence of all HIV-1 genes and RNA structural regions. APOBEC3G- or APOBEC3F-mediated hypermutation was identified using Hypermut 2.0 (Rose and Korber, 2000; Bruno et al., 2014). Sequences were checked for premature stop codons in all open reading frames. Proviruses were considered intact if they lacked deletions other than normal length polymorphisms and did not contain hypermutation or premature stop codons in open reading frames.

### Integration site-specific ddPCR

To quantify the frequency of specific proviruses in vivo, we used ddPCR (QX200; Bio-Rad) with a universal forward primer in a conserved region of the HIV 3′ LTR and a reverse primer representing a host sequence downstream of the 3′ HVJ. To prevent non-specific amplification of unrelated proviral sequences, a provirus-specific, fluorescently labeled probe was designed to anneal across the 3′ HVJ. Primer and probe sequences are shown in Table S6. PCR reactions were run with the following parameters: 95°C for 10 min, 95°C for 30 s, 56°C for 2 min (steps 2–3 for 50 cycles), 98°C for 10 min, hold at 4°C (temperature change rate: 2°C/s). Primers and probes were used at a final concentration of 900 and 250 nM, respectively. Specificity was tested first against whole genome-amplified DNA samples with and without the provirus of interest. Then, in vivo clone size was determined by measuring the frequency of proviral copies in a sample of resting memory CD4^+^ T cell–derived gDNA. To minimize the amount of gDNA consumed, proviruses were multiplexed such that two proviruses were counted in each reaction, one with a probe fluorescing in FAM and another in VIC. Using ddPCR for RPP30 as previously described, we counted cell equivalents in gDNA samples and normalized copies of proviral integration sites (Berry et al., 2016; Bruner et al., 2019).

### Statistics

Plating schemes were developed using Poisson statistics. For pairwise comparisons, a Wilcoxon test was performed.

### Study approval

The Johns Hopkins and University of Pennsylvania Institutional Review Boards approved this study. All participants provided written informed consent before enrollment in this study.

### Online supplemental material

Fig. S1 shows the sensitivity of the optimized HIV-1 virion detection method and depicts the experimental scheme and data generated using the RCAS internal standard. Fig. S2 shows the results of T cell stimulation experiments performed to optimize cellular stimulation, activation, and proliferation. Table S1 lists characteristics and biomedical data for each study participant. Table S2 contains HIV-1 reservoir measurements for each study participant. Table S3 contains a comparison of theoretical and actual infected cell plating frequencies. Table S4 contains a list of all 58 wells that had matched integration site, proviral type, proliferation, and virion production data. Table S5 lists all 11 wells that had matched in vivo clone size, integration site, provirus type, in vitro proliferation, and virion production data. Table S6 lists the primer and probe sequences used for integration site–specific ddPCR.

## Supplementary Material

Table S1 shows characteristics of study participants.

Table S2 shows initial IPDA results and plating scheme.

Table S3 shows observed and expected number of wells with proviruses.

Table S4 shows analysis of integration sites.

Table S5 shows in vivo clone frequency and ex vivo proliferation data for proviruses with the indicated integration sites.

Table S6 lists primers and probes used for integration site–specific ddPCR.

## Data Availability

All relevant data are available within the paper and supplementary materials, and upon request from the lead contact, R.F. Siliciano (rsiliciano@jhmi.edu). Further information and requests for reagents generated or used in this study are also available upon request from the lead contact.
