# Supplementary material for: CD4+ T cells with latent HIV-1 have reduced proliferative responses to T cell receptor stimulation
Source: J Exp Med. 2024 Jan 25;221(3):e20231511. doi: 10.1084/jem.20231511 (PMC10818065; doi:10.1084/jem.20231511)
Supplement: Table S1 — shows characteristics of study participants. [file JEM_20231511_TableS1.docx]

Table S1. **Characteristics of study participants**

| ID | Sex | Race^1^ | Age | Time since HIV diagnosis  (years) | Time on ART^2^  (years) | Current ART Regimen^3^ | Time with HIV RNA <LOD^4^ | CD4 Count^5^  (cells/μl) |
| --- | --- | --- | --- | --- | --- | --- | --- | --- |
| 012 | Male | AA | 44 | 22.6 | 17.5 | ABC/DTG/3TC/TDF | 12.7 | 513 |
| 017 | Male | AA | 49 | 11.9 | 11.6 | ETV/COBI/FTC/TAF | 11.5 | 508 |
| 021 | Male | AA | 59 | 19.8 | 15 | EFV/FTC/TDF | 7.1 | 973 |
| 024 | Male | AA | 61 | 28.8 | 20.8 | DTG/RPV | 16.3 | 362 |
| 040 | Male | AI/Mixed | 34 | 11.7 | 10.9 | ABC/DTG/3TC/TDF | 10.7 | 545 |
| 209 | Male | AA | 60 | 30 | 20.2 | BIC/FTC/TAF | ≥9.8 | 896 |
| 361 | Female | AA | 43 | 29 | 10 | ABC/DTG/3TC | 10 | 906 |
| 383 | Female | AA | 72 | 20.1 | 17.4 | FTC/RPV/TAF | 15.6 | 459 |
| 417 | Male | AA | 49 | 26.5 | 18.3 | EVG/COBI/FTC/TAF | 13.6 | 517 |
| 422 | Male | "other" | 55 | 21.3 | 19.3 | FTC/TAF/CAB | 14.7 | 700 |
| Median |  |  | 55 | 21.9 | 17.5 |  | 12.1 | 531 |

^1^AA = African American; AI = American Indian.

^2^Time since starting the first ART regimen.

^3^Antiretroviral drug abbreviations: ABC, abacavir; BIC, bictegravir; CAB, cabotegravir; COBI, cobicistat; DTG, dolutegravir; EFV, efavirenz; ETV, etravirine; EVG, elvitegravir; FTC, emtricitabine; RPV, rilpivirine; TAF, tenofovir alafenamide; TDF, tenofovir disoproxil fumarate; 3TC, lamivudine.

^4^Years of stable suppression with plasma HIV-RNA below LOD of clinical assays.

^5^CD4^+^ T cell count at time of sampling.
