# Supplementary material for: CD4+ T cells with latent HIV-1 have reduced proliferative responses to T cell receptor stimulation
Source: J Exp Med. 2024 Jan 25;221(3):e20231511. doi: 10.1084/jem.20231511 (PMC10818065; doi:10.1084/jem.20231511)
Supplement: Table S2 — shows initial IPDA results and plating scheme. [file JEM_20231511_TableS2.docx]

Table S2. **Initial IPDA results and plating scheme**

| **ID** | **Plating scheme^1^** | **Intact proviruses^2^**  **(/10^6^ resting memory CD4^+^ T cells)** | **3' defective**  **proviruses^2^**  **(/10^6^ resting memory CD4^+^ T cells)** | **5' defective**  **proviruses^2^**  **(/10^6^ resting memory CD4^+^ T cells)** | **Fraction intact** | **Total cells plated/**  **well** | **Expected infected cells/well** |
| --- | --- | --- | --- | --- | --- | --- | --- |
| 012 | 1 | 432 | 906 | 93 | 0.302 | 699 | 1 |
| 017 | 1 | 190 | 905 | 1161 | 0.084 | 443 | 1 |
| 040 | 1 | 145 | 134 | 50 | 0.440 | 3038 | 1 |
| 209 | 1 | 61 | 650 | 548 | 0.049 | 823^3^ | 1 |
| 417 | 1 | 225 | 1506 | 2210 | 0.057 | 282^3^ | 1 |
| 422 | 1 | 199 | 2192 | 1399 | 0.052 | 264 | 1 |
| 021 | 2 | 44 | 596 | 990 | 0.027 | 215 | 0.35 |
| 024 | 2 | 88 | 790 | 394 | 0.069 | 275 | 0.35 |
| 361 | 2 | 305 | 4357 | 1965 | 0.046 | 53 | 0.35 |
| 383 | 2 | 166 | 933 | 957 | 0.081 | 170 | 0.35 |
| median |  | 178 | 905 | 974 | 0.065 | 279 |  |

^1^See text for details.

^2^Based on IPDA analysis before dilution and plating.

^3^Values plated were ~10% higher than estimated for 1 infected cell/well.
