# Supplementary material for: CD4+ T cells with latent HIV-1 have reduced proliferative responses to T cell receptor stimulation
Source: J Exp Med. 2024 Jan 25;221(3):e20231511. doi: 10.1084/jem.20231511 (PMC10818065; doi:10.1084/jem.20231511)
Supplement: Table S3 — shows observed and expected number of wells with proviruses. [file JEM_20231511_TableS3.docx]

Table S3. **Observed and expected number of wells with proviruses**

|  | Plating Scheme 1  One infected cell/well | | | | Plating Scheme 2  Limit dilution | | | |
| --- | --- | --- | --- | --- | --- | --- | --- | --- |
| Number of infected cells/well | 0 | ≥1 | 1 | ≥2 | 0 | ≥1 | 1 | ≥2 |
| Theoretical fraction of wells (%)^1^ | 36.8 | 63.2 | 36.8 | 26.4 | 70.5 | 29.5 | 24.7 | 4.9 |
| Observed fraction of wells (%)^2^ | 33.2 | 66.8 | 45.6^3^ | 21.2^3^ | 70.8 | 29.2 | 26.2^3^ | 3.0^3^ |

^1^Based on Poisson statistics.

^2^Based on results from all donor samples plated at the indicated number of infected cells.

^3^Based on the number of unique provirus types observed (intact, 3′ defective, 5′ defective).
