# Supplementary material for: CD4+ T cells with latent HIV-1 have reduced proliferative responses to T cell receptor stimulation
Source: J Exp Med. 2024 Jan 25;221(3):e20231511. doi: 10.1084/jem.20231511 (PMC10818065; doi:10.1084/jem.20231511)
Supplement: Table S4 — shows analysis of integration sites. [file JEM_20231511_TableS4.docx]

Table S4. **Analysis of integration sites**

| Integration Site | Gene^2^ | Genic Region | Orientation | Provirus by IPDA (by NGS) ^3^ | Donor ID | Plate | Well | Cell Fold Increase^4^ | HIV Virions^5^ |
| --- | --- | --- | --- | --- | --- | --- | --- | --- | --- |
| chr1:170553255 | GORAB | Exon | Same | 5' Defective | 012 | P1 | C01 | 442 | 250353 |
| chr6:12131972 | HIVEP1 | Intron | Same | 5' Defective | 012 | P2 | D01 | 663 | ND |
| chr14:106145637 | IGH | Exon | Opposite | 5' Defective | 012 | P1 | E05 | 914 | ND |
| chr22:18734637 | LINC01662 | Intron | Same | 5' Defective | 012 | P2 | D11 | 2053 | ND |
| chr5:177856527 | LOC107986490 | Exon | Same | 3' Defective | 012 | P3 | E11 | 519 | ND |
| chr11:66119008 | PACS1 | Intron | Same | 3' Defective | 012 | P3 | F11 | 1397 | ND |
| chr11:119231129 | CBL | Intron | Same | Intact (INT) | 017 | P4 | E09 | 1060 | ND |
| chr16:50761688 | CYLD | Intron | Same | 5' Defective | 017 | P4 | G05 | 496 | ND |
| chr5:131562148 | RAPGEF6 | Intron | Opposite | 5' Defective | 017 | P4 | H06 | 1565 | ND |
| chr16:536974 | CAPN15 | Intron | Same | 5' Defective | 021 | P6 | D04 | 327 | ND |
| chr20:41581883 | CHD6 | Intron | Opposite | 5' Defective | 021 | P2 | E04 | 1371 | ND |
| chrX:45832100 | DS: LOC401585 (16 kb) | Intergenic | N/A | 3' Defective | 021 | P1 | D07 | 893 | ND |
| chr1:45665458 | GPBP1L1 | Intron | Opposite | 3' Defective | 021 | P1 | B03 | 412 | 2219 |
| chr14:106758527 | IGH | Exon | Opposite | 5' Defective | 021 | P6 | C05 | 523 | 2224 |
| chr1:149400960 | LOC100996717 | Intron | Same | 5' Defective | 021 | P6 | D08 | 357 | ND |
| chr17:6772814 | XAF1 | Intron | Same | 5' Defective | 021 | P3 | D05 | 3265 | 1048 |
| chr17:19954109 | AKAP10 | Intron | Opposite | 3' Defective | 024 | P8 | G12 | 730 | ND |
| chrX:13161569 | LOC105373134 | Intron | Opposite | 5' Defective | 024 | P2 | G02 | 2289 | ND |
| chr1:117498290 | MAN1A2 | Intron | Opposite | 3' Defective | 024 | P4 | B05 | 1449 | ND |
| chr16:68205803 | NFATC3 | Intron | Same | 5' Defective | 024 | P8 | F12 | 450 | ND |
| chr11:72053840 | NUMA1 | Intron | Opposite | 3' Defective | 024 | P8 | E08 | 501 | ND |
| chr16:29018501 | US: LAT (28 kb) | Intergenic | N/A | 3' Defective | 024 | P8 | D08 | 368 | ND |
| chr19:36719078 | ZNF850 | Exon | Same | Intact (HYP) | 040 | P1 | F03 | 40 | 4818 |
|  |  |  |  |  |  | P2 | C08 | 107 | 14978 |
|  |  |  |  |  |  |  | G02 | 58 | ND |
|  |  |  |  |  |  | P3 | D03 | 196 | 62322 |
|  |  |  |  |  |  |  | E08 | 54 | ND |
|  |  |  |  |  |  | P4 | F09 | 54 | 2423622 |
|  |  |  |  |  |  |  | G12 | 88 | 7680228 |
|  |  |  |  |  |  |  | H07 | 53 | 187287 |
|  |  |  |  |  |  |  | H08 | 218 | 23420 |
| chr5:157279530 | CYFIP2 | Intron | Same | 3' Defective | 209 | P2 | C09 | 1293 | ND |
| chr19:52300428 | ZNF480 | Exon | Same | 3' Defective | 209 | P2 | G05 | 3122 | ND |
| chr1:193234083 | CDC73 | Intron | Same | 3' Defective | 361 | P8 | B08 | 726 | ND |
| chr17:37628598 | DDX52 | Exon | Opposite | 5' Defective | 361 | P7 | A05 | 3395 | 923 |
| chr12:132700266 | PXMP2 | Intron | Same | 3' Defective | 361 | P4 | B05 | 1671 | ND |
| chr19:46929703 | ARHGAP35 | Intron | Same | 3' Defective | 383 | P1 | B07 | 1470 | ND |
| chr18:2727453 | SMCHD1 | Intron | Same | 5' Defective | 383 | P1 | A04 | 1251 | 449 |
| chr17:77233893 | US: SEC14L1 (17 kb) | Intergenic | N/A | 3' Defective | 383 | P3 | F09 | 2667 | 5304 |
|  |  |  |  |  |  |  | G06 | 310 | ND |
| chr12:62295112 | USP15 | Intron | Same | 5' Defective | 383 | P1 | D02 | 742 | ND |
| chr16:3529125 | CLUAP1 | Intron | Same | 5' Defective | 417 | P1 | F01 | 8613 | 2456 |
| chr11:67161828 | KDM2A | Intron | Same | 3' Defective | 417 | P1 | C12 | 329 | 3014 |
| chr22:38906018 | LOC105373032 | Exon | Same | 5' Defective | 417 | P1 | D08 | 2690 | 1270 |
| chr18:2665558 | SMCHD1 | Intron | Same | 3' Defective | 417 | P2 | B02 | 523 | ND |
| chr15:44643388 | SPG11 | Intron | Opposite | 5' Defective | 417 | P2 | A10 | 552 | ND |
| chr5:146500698 | TCERG1 | Intron | Same | 3' Defective | 417 | P1 | D01 | 3058 | ND |
| chr11:95624559 | US: LOC105369441 (9 kb) | Intergenic | N/A | 3' Defective | 417 | P1 | D06 | 551 | ND |
| chr16:89354166 | ANKRD11 | Intron | Opposite | 3' Defective | 422 | P3 | H03 | 482 | ND |
| chr8:61552712 | ASPH | Intron | Opposite | 5' Defective | 422 | P3 | H04 | 344 | 16073 |
| chr10:102967192 | CNNM2 | Intron | Same | 5' Defective | 422 | P3 | A03 | 374 | ND |
| chr7:150465172 | GIMAP8 | Intron | Same | 3' Defective | 422 | P3 | C10 | 390 | ND |
| chr17:63840663 | SMARCD2 | Intron | Opposite | 3' Defective | 422 | P3 | F01 | 1036 | ND |
| chr16:29541351 | SMG1P2 | Intron | Opposite | 5' Defective | 422 | P3 | C07 | 1421 | ND |
| chr19:39452013 | SUPT5H | Intron | Same | 5' Defective | 422 | P3 | C06 | 508 | ND |
|  |  |  |  |  |  |  | F07 | 1658 | ND |
| chr10:58355464 | UBE2D1 | Intron | Same | 3' Defective | 422 | P3 | H02 | 1654 | ND |
| chr10:37844255 | ZNF248 | Intron | Opposite | Intact (HYP) | 422 | P4 | H02 | 6215 | ND |

^1^Based on hg38. chr = chromosome.

^2^US or DS indicate integration site was upstream or downstream, respectively, of the indicated gene by the indicated distance.

^3^Provirus type by Intact Proviral DNA Assay (Bruner et al., 2019). Near full-length proviral sequencing result shown in parentheses. HYP = hypermutated provirus.

^4^*Ex vivo* clonal expansion over one week starting from a single infected cell.

^5^*Ex vivo* virion production over one week starting from a single infected cell.

**References**

Bruner, K.M., Z. Wang, F.R. Simonetti, A.M. Bender, K.J. Kwon, S. Sengupta, E.J. Fray, S.A. Beg, A.A.R. Antar, K.M. Jenike, et al. 2019. A quantitative approach for measuring the reservoir of latent HIV-1 proviruses. *Nature*. 566:120–125. 10.1038/s41586-019-0898-8
