# Supplementary material for: CD4+ T cells with latent HIV-1 have reduced proliferative responses to T cell receptor stimulation
Source: J Exp Med. 2024 Jan 25;221(3):e20231511. doi: 10.1084/jem.20231511 (PMC10818065; doi:10.1084/jem.20231511)
Supplement: Table S5 — shows in vivo clone frequency and ex vivo proliferation data for proviruses with the indicated integration sites. [file JEM_20231511_TableS5.docx]

Table S5. **In vivo clone frequency and ex vivo proliferation data for proviruses with the indicated integration sites**

| Integration Site^1^ | Gene^2^ | Provirus by IPDA (by NGS)^3^ | Donor  ID | In vivo frequency  (copies/10^6^ resting  CD4^+^ T cells)^4^ | LOD  (copies/10^6^ resting  CD4^+^ T cells)^5^ | Fraction of Reservoir^6^ (%) | Culture Wells^7^ | Fold Increase  In Infected Cells^8^ | HIV Virions^9^ |
| --- | --- | --- | --- | --- | --- | --- | --- | --- | --- |
| chr19:36719078 | ZNF850 | Intact (HYP) | 40 | 94.87 | 2.08 | 28.84 | 9 | 58 | 23420 |
| chr19:39452013 | SUPT5H | 5' defective | 422 | 41.86 | 2.03 | 1.1 | 2 | 1083 | 0 |
| chr11:67161828 | KDM2A | 3' defective | 417 | 22.82 | 7.72 | 0.58 | 1 | 329 | 3014 |
| chr16:68205803 | NFATC3 | 5' defective | 24 | 9.76 | 8.09 | 0.77 | 1 | 450 | 0 |
| chrX:13161569 | LOC105373134 | 5' defective | 24 | 9.76 | 8.09 | 0.77 | 1 | 2289 | 0 |
| chr17:6772814 | XAF1 | 5' defective | 21 | 6.26 | 0.88 | 0.38 | 1 | 3265 | 1048 |
| chr17:77233893 | US: SEC14L1 (17 kb) | 3' defective | 383 | 2.55 | 2.55 | 0.12 | 2 | 1488 | 2652 |
| chr10:37844255 | ZNF248 | Intact (HYP) | 422 | 2.25 | 2.03 | 0.06 | 1 | 6215 | 0 |
| chr16:3529125 | CLUAP1 | 5' defective | 417 | Undetected | 7.72 | Undetected | 1 | 8613 | 2456 |
| chr5:146500698 | TCERG1 | 3' defective | 417 | Undetected | 7.72 | Undetected | 1 | 3058 | 0 |
| chr1:149400960 | LOC100996717 | 5' defective | 21 | Undetected | 0.88 | Undetected | 1 | 357 | 0 |

^1^Based on hg38. chr = chromosome

^2^US or DS indicate integration site was upstream or downstream, respectively, of the indicated gene by the indicated distance.

^3^Provirus type by Intact Proviral DNA Assay (Bruner et al., 2019). Near full-length proviral sequencing result shown in parentheses. HYP = hypermutated provirus.

^4^Number of proviral copies per million resting memory CD4^+^ T cells in vivo.

^5^Limit of detection, calculated using the number of resting memory CD4^+^ T cell equivalents screened.

^6^Fraction of cells in the reservoir that have the indicated integration site.

^7^Number of culture wells containing cells with the indicated integration site.

^8^Ex vivo clonal expansion over one week starting from a single infected cell.

^9^Ex vivo virion production over one week starting from a single infected cell

**References**

Bruner, K.M., Z. Wang, F.R. Simonetti, A.M. Bender, K.J. Kwon, S. Sengupta, E.J. Fray, S.A. Beg, A.A.R. Antar, K.M. Jenike, et al. 2019. A quantitative approach for measuring the reservoir of latent HIV-1 proviruses. *Nature*. 566:120–125. 10.1038/s41586-019-0898-8
