# Supplementary material for: CD4+ T cells with latent HIV-1 have reduced proliferative responses to T cell receptor stimulation
Source: J Exp Med. 2024 Jan 25;221(3):e20231511. doi: 10.1084/jem.20231511 (PMC10818065; doi:10.1084/jem.20231511)
Supplement: Table S6 — lists primers and probes used for integration site–specific ddPCR. [file JEM_20231511_TableS6.docx]

Table S6. **Primers and probes used for integration site–specific ddPCR**

| **ID** | **Gene** | **Reverse Primer^1^** | **Probe** | **Fluor^2^** |
| --- | --- | --- | --- | --- |
| 012 | CDK6 | CTTATTTCCAAAGAGGGAAGC | CTCTAGCAATGAGCCAAACATTCCTTGG | VIC |
| 021 | LOC100996717 | CTTTGACCTAATGGATAAATGCC | CAGTGTGGAAAATCTCATCTCAGCAGTGAGG | VIC |
|  | XAF1 | GGAGGGACAGATGGGAGAA | CTCTAGCAGAAACCCTACAAACCAAACAGTGTG | FAM |
| 024 | NFATC3 | CTAGTTTATACTTTGCATTTCCCATG | CTCTAGCAGTTACAGTATAGAATAATCTCTCAAG | FAM |
|  | LOC105373134 | CTCTACACATCAAGGAGCAGAT | CTCTAGCAGAGTGTCAGAACTGAATTAGAG | VIC |
| 040 | ZNF850 | TTCAAGGAACACCAAGAGAAGT | CGGTGTGGAAAATCTCTAGCAGTAAATGTC | FAM |
| 383 | SEC14L1-US^3^ | ATTTACTTCTCGCTCACGCTAA | CAGTGTGGAAAATCTCTAGCACTCTGGAC | FAM |
| 417 | CLUAP1 | TCCCTCAATATCTGCGAAGAAC | CTCTAGCAGTTACATGAAAAGGACTTGAAC | VIC |
|  | TCERG1 | GACGGTAAAGTCATTCTCACAGTA | CAGTACGAAAAATCTCTAGCAAAAACTAGTGGT | FAM |
|  | KDM2A | CTCATTCTCTAATAAGTGTAGACTTTCC | CTCTAGCAGTTCTAGAAACCTCTACTATACTTC | VIC |
| 422 | ZNF248 | TTCTGTAGGGCAGGGTTTATG | CTCTAGCATAAACCTGGCAAATTGATCC | VIC |
|  | SUPT5H | ATCATTTCTCCAACCCATCCTT | CTCTAGCAGGTAAGGATCCAGGTGTTAGAG | FAM |

¹The forward primer CTGTTGTGTGACTCTGGTAACT was used in all reactions.

^2^The quencher MGBNFQ was used in all probes.

^3^US indicates that the integration site was upstream of SEC14L1 by 17 kb.
